# Environmental DNA recovers fish composition turnover of the coral reefs of West Indian Ocean islands

**DOI:** 10.1002/ece3.11337

**Published:** 2024-05-16

**Authors:** Mélissa Jaquier, Camille Albouy, Wilhelmine Bach, Conor Waldock, Virginie Marques, Eva Maire, Jean Baptiste Juhel, Marco Andrello, Alice Valentini, Stéphanie Manel, Tony Dejean, David Mouillot, Loïc Pellissier

**Affiliations:** ^1^ Ecosystems and Landscape Evolution, Institute of Terrestrial Ecosystems, Department of Environmental Systems Science ETH Zürich Zürich Switzerland; ^2^ Unit of Land Change Science Swiss Federal Research Institute WSL Birmensdorf Switzerland; ^3^ CEFE, Univ. Montpellier, CNRS, EPHE‐PSL University, IRD Montpellier France; ^4^ MARBEC, Univ. Montpellier, CNRS, Ifremer, IRD Montpellier France; ^5^ Lancaster Environment Centre Lancaster University Lancaster UK; ^6^ SPYGEN Le Bourget‐du‐Lac France; ^7^ Institut Universitaire de France Paris France

**Keywords:** biodiversity, biomonitoring, coral reef, diffusion, environmental DNA, scattered islands

## Abstract

Islands have been used as model systems to study ecological and evolutionary processes, and they provide an ideal set‐up for validating new biodiversity monitoring methods. The application of environmental DNA metabarcoding for monitoring marine biodiversity requires an understanding of the spatial scale of the eDNA signal, which is best tested in island systems. Here, we investigated the variation in Actinopterygii and Elasmobranchii species composition recovered from eDNA metabarcoding along a gradient of distance‐to‐reef in four of the five French Scattered Islands in the Western Indian Ocean. We collected surface water samples at an increasing distance from reefs (0 m, 250 m, 500 m, 750 m). We used a metabarcoding protocol based on the ‘teleo’ primers to target marine reef fishes and classified taxa according to their habitat types (benthic or pelagic). We investigated the effect of distance‐to‐reef on β diversity variation using generalised linear mixed models and estimated species‐specific distance‐to‐reef effects using a model‐based approach for community data. Environmental DNA metabarcoding analyses recovered distinct fish species compositions across the four inventoried islands and variations along the distance‐to‐reef gradient. The analysis of β*‐*diversity variation showed significant taxa turnover between the eDNA samples on and away from the reefs. In agreement with a spatially localised signal from eDNA, benthic species were distributed closer to the reef than pelagic ones. Our findings demonstrate that the combination of eDNA inventories and spatial modelling can provide insights into species habitat preferences related to distance‐to‐reef gradients at a small scale. As such, eDNA can not only recover large compositional differences among islands but also help understand habitat selection and distribution of marine species at a finer spatial scale.

## Graphical abstract

This study utilised environmental DNA metabarcoding to examine the variation in fish species composition in the Western Indian Ocean's French Scattered Islands across different distances from reefs. The results revealed distinct species composition and significant turnover of taxa between eDNA samples near and away from reefs, suggesting a spatially localised eDNA signal. The findings highlight the potential of eDNA metabarcoding for understanding species habitat preferences and distribution at finer spatial scales.

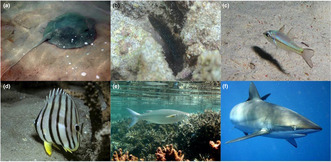

## 1 INTRODUCTION

Islands have been used as model systems to explore a large array of biological and ecological processes for the past two centuries (e.g., Jacquet et al., 2017; Nogué et al., 2021; Whittaker & Fernández‐Palacios, 2007). The peculiarity of islands lies in the interaction between biogeographical and oceanographical conditions and their past environments, which produced diverse and endemic biotic assemblages (Dietzel et al., 2021; Stuart et al., 2012). For example, islands harbour 20% of all terrestrial species while covering only 3.5% of Earth's land area (Kier et al., 2009; Whittaker et al., 2017). Island coral reefs likewise have exceptional diversity (Cinner et al., 2018; Hoegh‐Guldberg, 2011), while also being among the most vulnerable ecosystems to future climate conditions (Pandolfi et al., 2011). As such, coral reefs on islands need efficient methods that can be applied rapidly to monitor changes in their associated biodiversity. More broadly, islands and their coral reefs could represent natural observatories to better understand changing biodiversity events using novel monitoring techniques (Kueffer et al., 2014).

Environmental DNA (eDNA) metabarcoding is efficient in gathering biodiversity data and is easier to deploy on isolated islands than traditional sampling (Juhel et al., 2020; Pawlowski et al., 2020). eDNA represents the genetic material released by organisms into their surrounding environment and obtained from environmental samples without isolating the individuals (Taberlet et al., 2018). An eDNA sample is characterised by a complex mixture of intracellular and extracellular DNA molecules that vary in their stage of degradation. Environmental and physico‐chemical factors (e.g., water temperature, chemistry and microbial activity) can influence the persistence of eDNA molecules in the environment, their concentration and thus their detectability (Goldberg et al., 2016; Harrison et al., 2019; Jo et al., 2020). For example, the production and shedding rate of DNA material from fish is likely to vary with the metabolism rate, behaviour, age, size, sex and taxon, as well as with season and habitat characteristics (Barnes & Turner, 2015; Thalinger et al., 2021). Despite this underlying variability, an increasing number of studies have documented the reliability of this technique in recovering information on marine biodiversity (e.g., Boulanger et al., 2021; Fediajevaite et al., 2021; McElroy et al., 2020). For example, when comparing eDNA metabarcoding to underwater visual census methods (UVC), Boussarie et al. (2018) and Polanco Fernández, Marques, et al. (2021) found that a large fraction of the species detected by eDNA in tropical marine reefs were not detected using traditional survey methods (e.g., UVC, baited remote underwater video). Given that eDNA is transported in the environment, it is necessary to know what volume of water a sample represents to reach the full potential of this sampling method in marine environments and perform accurate monitoring. Since eDNA molecules can be passively transported by sea currents, it is crucial to evaluate their ability to spread when using a recovered eDNA signal to assess species presence in space and time (Barnes & Turner, 2015).

Compared with freshwater systems, eDNA transport in the marine environment can be affected by additional factors that can lead to faster dispersion and lower DNA concentrations, which can impact the representativeness of the recovered signal (Thomsen et al., 2012). However, a growing number of studies have demonstrated the precise detection capability of eDNA metabarcoding, which makes this method well‐suited to monitor local assemblages of coral reef fishes (West et al., 2020) and endemic communities of remote islands (Roberts et al., 2002) that are under anthropogenic threats (Kreft et al., 2008; Nogué et al., 2021; Whittaker & Fernández‐Palacios, 2007). Yet, a larger water‐volume to biomass ratio at sea (Thomsen et al., 2012) could limit our ability to recover regional biodiversity at a large spatial scale (but see Mathon et al., 2022). Moreover, seawater salinity affects eDNA preservation and eDNA could be diluted during transport, which could be advantageous because faster degradation leads to more localised eDNA detection (Harrison et al., 2019). In line with this, a highly localised spatial resolution of eDNA in marine environments has been reported frequently (e.g., Minamoto et al., 2017; O'Donnell et al., 2017; Port et al., 2016; Rozanski et al., 2022; Stat et al., 2019), despite the potential for oceanic currents, eddies and waves to disperse eDNA over long distances (Barnes & Turner, 2015). By virtue of their isolation and simplified shoreline gradients, islands can be excellent model systems to better understand the diffusion of the eDNA signal.

The outlying French Scattered Islands, located in the Western Indian Ocean (WIO), represent a particularly suitable system to study the spatial scale of eDNA signals. These territories are surrounded by deep waters and are remarkably isolated, making them ideal to study diffusion of eDNA in natural coral reef conditions with little influence from nearby systems. Previous studies have demonstrated the exceptionally pristine status of their coral reefs, with abundant large fish and predator occurrences (Bigot et al., 2019; Chabanet & Durville, 2007), which, together with the prevalence of currents and eddies, indicates their potential as a biodiversity hotspot (Bigot et al., 2019; Quod et al., 2007). Furthermore, these isolated islands are inhabited only by small military detachments, making them appropriate locations to investigate biodiversity with little direct anthropogenic influence (Conand et al., 2013). Nevertheless, due to its proximity to Madagascar, Juan De Nova Island is vulnerable to poaching, especially of sea cucumbers (Conand et al., 2015), and its coral reefs were damaged by the IDAI tropical storm in March 2019. Thus, the Scattered Islands are of great conservation priority (O'Donnell et al., 2017), and there is a pressing need for non‐invasive and efficient whole‐ecosystem surveys.

Here, we investigated the spatial eDNA signal along an environmental distance‐to‐reef gradient from inshore (reef) to farther offshore (pelagic) across four reef systems in the Scattered Islands. We first compared the capabilities of eDNA metabarcoding and traditional surveys in detecting species from benthic and pelagic habitats. We then studied the ability of eDNA to detect a decay of similarity between communities separated by increasing geographical distances. This decrease in the eDNA signal from the reef to the pelagic zone should be captured under the hypothesis that eDNA transport (diffusion) is limited to short distances (Cantera et al., 2019). Lastly, we examined species‐specific responses to environmental covariates, as eDNA production, amount and detectability may vary between species (Buxton et al., 2017; Pilliod et al., 2014; Thalinger et al., 2021).

## 2 MATERIALS AND METHODS

### 2.1 Study area

**FIGURE 1:**
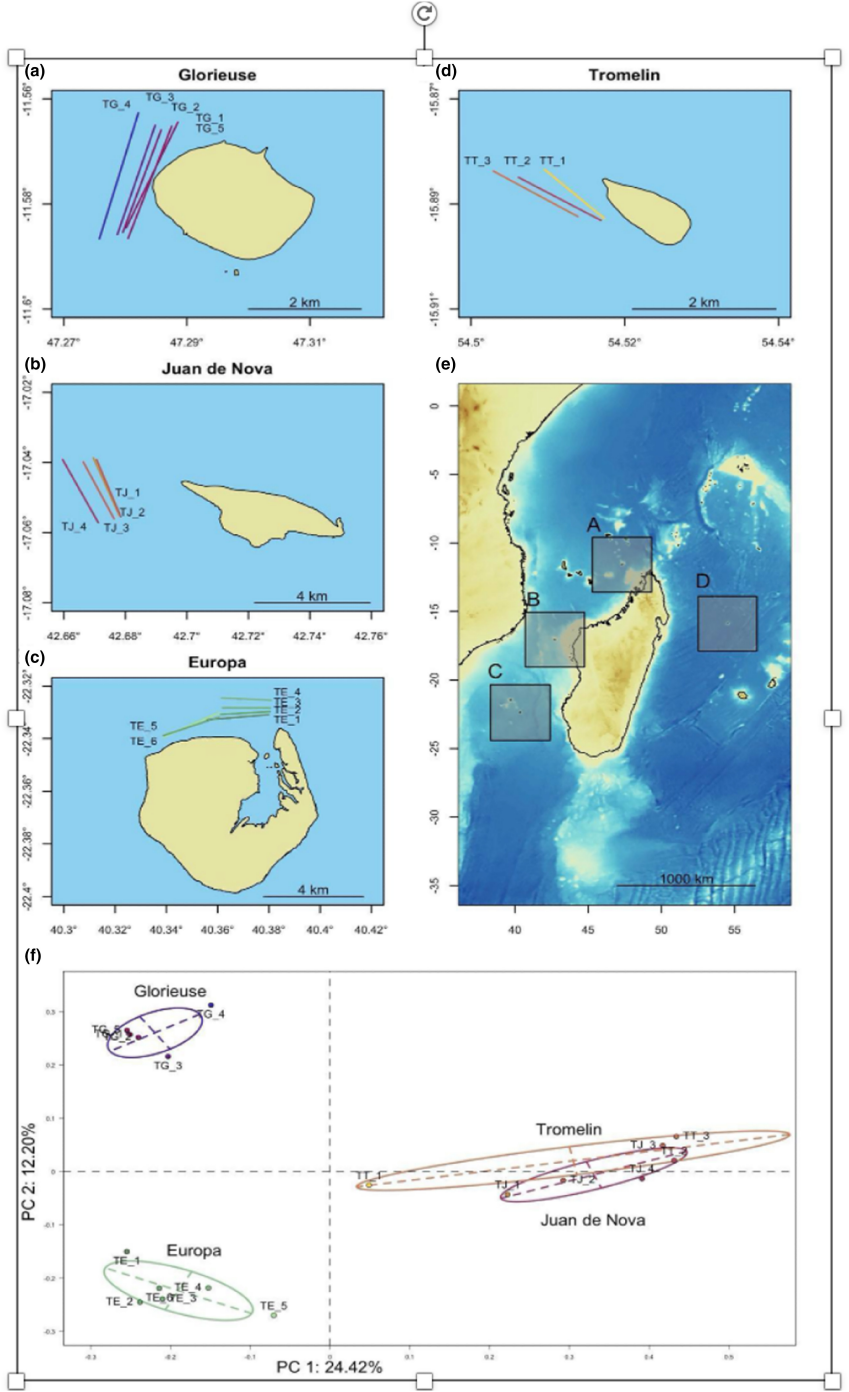
Area of environmental DNA (eDNA) sampling across (e) the French Scattered Islands, in (a) Glorieuse, (b) Juan de Nova, (c) Europa and (d) Tromelin. The lines indicate the transects filtered at each island. (f) Compositional differences (principal coordinates analysis, PCoA) from the molecular operational taxonomic unit (MOTU) presence‐absence matrix between eDNA sampling transects. Transect colours in (a) to (d) correspond to the transect positions in the ordination space (f).

All four of the studied islands, Europa, Juan de Nova, Grande Glorieuse (further referred to as Glorieuse) and Tromelin, belong to the Scattered Islands, situated around Madagascar in the WIO (Figure 1a–e). These islands feature an emerging land mass on a reef, whereas the fifth Scattered Island, Bassas da India, is a sub‐circular atoll rim covered by the sea at high tide and was therefore excluded from this study. Together, the four studied oceanic islands present two major types of reef structure: atolls (Europa) and banks (Glorieuse, Juan de Nova and Tromelin; Andrefouet et al., 2006, 2009), covering a total coral reef area of 406.4 km^2^. All of the islands have a forereef with a gradient from a typical coral reef habitat to a pelagic zone. Juan de Nova is the island with the greatest reef area, followed by Glorieuse, Europa and Tromelin, while Europa has the largest land area, followed by Glorieuse, Juan de Nova and Tromelin (Quod et al., 2007). Glorieuse is located at the northern entry point of the Mozambique channel, a region where high mixing of waters prevails with considerable connectivity and retention of larvae, resulting in high species richness (Obura, 2012). Tromelin Island is the most isolated geographically, and its climatic conditions are hostile, with intense winds and frequent hurricanes. Approximately four times per year, strong eddies develop within the Mozambique channel (Schouten et al., 2003), which increases retention rates of larvae, thus reinforcing the genetic structure among the islands (O'Donnell et al., 2017). This is especially true for Juan de Nova, located in the narrowest part of the channel, where eddies boost local retention of species with a short pelagic larval duration (O'Donnell et al., 2017).

### 2.2 Environmental DNA data collection

We collected a total of 36 water samples along 18 transects at the four islands (Europa = 6; Glorieuse = 5; Juan de Nova = 4; Tromelin = 3), from 8 April to 28 April 2019. For the eDNA sampling, we applied a large volume sampling method using dedicated filtration capsules, which makes it possible to integrate a large area and accurately represent the biodiversity (Govindarajan et al., 2022). We performed two filtration replicates in parallel, one on each side of the boat, positioning the entry of the tubes a few cm below the surface and undertaking a 30‐min transect filter of about 30 L in total. We performed the first transect of each island close to the shore on shallow reefs. We then conducted transects at different distances from the initial transect for each island based on the observed changes in depth. For Europa, we conducted transects at 50, 300 and 600 m from the initial transect, due to a gradual decrease in depth extending from the northern part of the island. By contrast, depth decreased rapidly from the initial transect in Glorieuse and Tromelin; we performed transects at 50, 100 and 300 m from the first transect for Glorieuse, but at only 50 and 100 m for Tromelin, given the small size of this island, the sudden decrease in depth and safety considerations (Figure 1a–d). We filtered the eDNA samples in situ. The filtration gear was composed of an Athena® peristaltic pump (Proactive Environmental Products LLC, Bradenton, Florida, USA; nominal flow of 1.0 L min^−1^), a VigiDNA® 0.2 μM cross flow filtration capsule (SPYGEN, le Bourget du Lac, France) and disposable sterile tubing for each filtration capsule. After filtration, capsules were emptied of water, filled with 80 mL of CL1 conservation buffer (SPYGEN, le Bourget du Lac, France) and stored at room temperature in the dark. We followed a rigorous protocol to avoid contamination during fieldwork, using disposable gloves and single‐use filtration equipment to process each water sample.

We performed DNA extraction, amplification and high‐throughput sequencing following the protocol of Polanco Fernández, Marques, et al. (2021), which is further detailed in Appendix S1. We amplified DNA fragments by polymerase chain reaction (PCR) with the ‘teleo’ primer (forward: ACACCGCCCGTCACTCT, reverse: CTTCCGGTACACTTACCATG; Valentini et al., 2016) that amplify a region of 64 base pairs on average (range 29–96 bp) of the mitochondrial 12S region, designed to capture both teleost and Elasmobranchii taxa (Polanco Fernández, Marques, et al., 2021). We performed library preparation and sequencing at Fasteris (Geneva, Switzerland). Specifically, we prepared four libraries using the MetaFast protocol (a ligation‐based method) and sequenced them separately. We carried out paired‐end sequencing using a MiSeq sequencer (2 × 125 bp, Illumina, San Diego, CA, USA) on two MiSeq Flow Cell Kits (v3; Illumina), following the manufacturer's instructions. We ultimately used *n*_*y*_ = (2 × 18) − 1 = 35 samples, as we discarded one sample due to sequencing issues.

During these three laboratory steps, we applied a meticulous contamination control protocol (Valentini et al., 2016). Specifically, we performed DNA extraction, amplification and high‐throughput sequencing in distinct dedicated rooms set up with positive air pressure, UV treatment and frequent air renewal and we dressed in full protective clothing before entering a room. We amplified two negative extraction controls and one negative PCR control of ultrapure water (12 replicates) and sequenced them in parallel to the samples. We did not detect any contamination in these extraction and PCR controls.

### 2.3 Bioinformatic pipelines

We analysed sequencing outputs using two distinct bioinformatic pipelines: one based on the OBITools toolkit (Boyer et al., 2016), hereafter called the species pipeline, and the other based on the SWARM clustering algorithm (Mahé et al., 2015), hereafter called the MOTU pipeline (Appendix S1, Figure S1). MOTUs (molecular operational taxonomic units) represent interpretable discrete taxonomic units expected to be equivalent to species (Pellissier et al., 2014; Sales et al., 2021). The two analysis workflows are complementary: the species pipeline enables identification at the species level, for comparison with the species recovered with traditional monitoring, whereas the MOTU pipeline can estimate the number of species (globally and by clade) present in the case of an incomplete reference database, like for tropical coral reefs. MOTUs are not as suitable for fine species‐level assignment, as very closely related species might cluster together due to genetic proximity and be missed in the final inventory (Marques et al., 2020), but they are useful for some biodiversity analyses because species identity is not required.

Following Valentini et al. (2016), for the species pipeline we used OBITools to merge sequencing outputs, then demultiplex, clean and assign sequences to a taxonomy. We merged forward and reverse reads using *illuminapairedend*, then demultiplexed sequences (i.e., assigned them to each sample) using *ngsfilter and obisplit*. We then analysed each sample independently before pooling the taxa list for final ecological analysis. We de‐replicated the sequences using *obiuniq*, and we removed sequences smaller than 20 bp or present in <10 reads using *obigrep*. We cleaned the sequences using *obiclean* with the default threshold (0.05) to discard sequences likely originating from PCR or sequencing errors.

Applying the MOTU bioinformatic pipeline from Marques et al. (2020), we used the SWARM algorithm to perform sequence clustering. We applied stringent abundance‐based threshold filtering to analyse the sequences in a way that did not depend on the completeness of the reference database. SWARM groups multiple sequences into sequence clusters (MOTUs) based on sequence similarity and abundance (Mahé et al., 2015). We used *vsearch* to merge paired‐end sequencing outputs (Rognes et al., 2016), *cutadapt* for demultiplexing and primer trimming (Martin, 2011) and *uchime* to identify chimeras. To generate clusters, we ran SWARM with the lowest d value (1), which is the minimum distance of two mismatches between each cluster's representative sequence. Once MOTUs were produced, we took the most abundant sequence within each cluster as the representative sequence for taxonomic assignment. To curate the data, we used the LULU post‐clustering curation algorithm (Frøslev et al., 2017). We applied stringent filters to discard potential PCR or sequencing errors and non‐specific amplifications, eliminating non‐specific amplifications (non‐fish), sequences detected in only one PCR replicate in the full dataset, sequences detected in <10 reads per occurrence and sequences identified as chimeras.

For both pipelines, we assigned the taxonomy of the sequences using the *ecotag* program with the EMBL genetic reference database, which includes 16,128 sequences from 10,546 species across all organisms (European Molecular Biology Laboratory, <www.ebi.ac.uk>, v141, downloaded in January 2020; Baker et al., 2000). We then curated the taxonomic assignments further and validated the *ecotag* outputs only if the identification match was 100% at the species level, 90%–99% at the genus level and 85%–90% at the family level (Marques et al., 2020). We included this step to be more conservative and to avoid overconfidence resulting from the lower common ancestor algorithm from *ecotag*, where species‐level assignment can happen even with an imperfect match. This step is meant as a correction and curation of existing ecotag outputs to downgrade potential assignments to be more conservative. To account for the incorrect assignment of sequences to samples due to tag‐jumps (Schnell et al., 2015), we excluded sequences from both pipelines with a frequency of occurrence <0.001 per taxon (or MOTU) and per library. We further corrected for index‐hopping (MacConaill et al., 2018) with empirically determined thresholds for each sequencing batch using experimental blanks (combinations of tags not present in the libraries) and applied them to each plate position across different libraries sequenced in the same batch.

### 2.4 Comparison of species lists between islands

After the taxonomic assignment of sequences in both pipelines, we cross‐checked the taxonomic identifications from eDNA against local fish faunal lists (comm. pers. Terres Australes et Antarctiques Françaises, TAAF) when possible, or using the Fishbase database (Froese & Pauly, 2021; Appendix S1). The TAAF started to record species presences in 1998 in Glorieuse, using underwater visual censuses (UVCs) or a free underwater course during dives for other measurements, where an unlisted fish was logged when encountered. The inventories have been updated continuously since then.

To explore the differences between islands in the MOTUs and taxa detected per eDNA sample, we performed a non‐parametric Kruskal‐Wallis analysis of variance (ANOVA) followed by a Dunn test with a Bonferroni correction. We additionally performed a principal coordinates analysis (PCoA) based on a Jaccard distance matrix to visually represent the main compositional differences between the eDNA samples collected across the four islands (Figure 1f). We implemented this PCoA using the *ade4* package (v.1.7.16; Chessel et al., 2004) in the R statistical programming environment (v.4.0.2; R Core Team, 2021).

### 2.5 Species habitat classifications

For the outputs from both pipelines, we used Fishbase to classify fish species, genera and families according to their preferences for two different habitat types when possible: benthic (category grouping benthic and demersal taxa that mostly live within the coral reefs and are non‐migratory) and pelagic (category comprising taxa that mostly live farther away from the reef and those that are migratory). For higher‐level assignments (genus and family), we chose the most widespread trait within the species of that clade. Of the 356 MOTUs recovered with the MOTU pipeline using sequence clustering, we assigned 199 MOTUs as benthic and 76 as pelagic, leaving 81 MOTUs unassigned to a habitat category due to insufficient taxonomic information. Of the 846 taxa recovered from the local inventory, we classified 704 taxa as benthic and 142 as pelagic. We compared the proportion of taxa from each habitat type between the faunal list and the recovered eDNA species list from the species pipeline and from the MOTU pipeline. We explored whether the habitat type influenced the detection of eDNA, i.e., if similar proportions of benthic and pelagic taxa were captured with eDNA compared with the faunal list based on traditional methods (UVC and individual observations). To perform all further analyses, we used the outputs from the MOTU pipeline, in which sequences were clustered into MOTUs, to ensure that the analyses did not depend on the coverage of the reference database (Marques et al., 2020).

### 2.6 Compositional changes with increasing distance from the reef

To analyse the change in eDNA composition from the reefs to the pelagic habitat, we computed the compositional differences in eDNA MOTUs (i.e., β‐diversity) between the samples from the first transect on the reef (as the reference) and the remaining samples taken farther away. This analysis accommodated our study design with a distance gradient from the reef for each island. We computed compositional differences at the level of the sample (filtration capsule), where the first transect had two reference samples and each other sample was compared to both of these reference samples. We calculated a distance matrix between samples based on differences in MOTU composition by computing the Jaccard dissimilarity index (*β*_jac_; Anderson et al., 2011) with the R package *betapart* v.1.5.2 (Baselga & Orme, 2012). This index is expressed as:

(1) $$\beta_{\mathrm{jac}}=\left(b+c\right)/\left(a+b+c\right)$$

where *a* is the number of shared MOTUs between two samples, *b* is the number of MOTUs unique to the first sample and *c* is the number of MOTUs unique to the second sample. The *β*_jac_ index ranges from 0, indicating an identical MOTU composition between samples, to 1, indicating a completely different MOTU composition between samples. In total, we computed 54 *β*_jac_ values: 12 for Jan de Nova, 14 for Europa, 10 for Tromelin and 18 for Glorieuse.

We quantified how much the composition changed with increasing distance from the reef. Specifically, we analysed the relationship between MOTU composition pairwise similarity (S = 1 − D, where S is the similarity and D is the *β*_jac_ dissimilarity; Koleff et al., 2003) and geographical distance between samples among the transects. We fitted Gaussian generalised linear mixed models (GLMM) to assess the effect of spatial distance and island identity on composition similarity. We accounted for the non‐independent pairs of samples in the study design by including a random effect for transect in the models. We fitted the models on the 54 similarity values and tested only the effects of distance and island identify (two variables), thus respecting the general statistical rule of 10 observations per predictor variable (e.g., Peduzzi et al., 1996). We ran three models, one considering all species, one considering only benthic/demersal species and one considering only pelagic species. We fitted GLMMs with the ‘MCMCglmm’ function in the R package *MCMCglmm* (Hadfield, 2010).

### 2.7 Hierarchical modelling of communities

We further assessed the distribution of the MOTUs along the gradient of distance from the reefs using hierarchical modelling of species communities (HMSC; Ovaskainen et al., 2017; Ovaskainen & Abrego, 2020). HMSCs are joint species distribution models (JSDMs; Warton et al., 2015), which include a hierarchical layer asking how species responses to environmental covariates depend on species traits and phylogenetic relationships (Abrego et al., 2017). We used the approach of spatially structured latent variables proposed by Ovaskainen et al. (2017). The data comprised occurrences of 356 MOTUs. We excluded MOTUs that had fewer than five occurrences in the samples, resulting in *n*_*s*_ = 164 MOTUs. As sampling units, we used the distinct samples of each transect (*n*_*y*_ = 35). As the response variable (the Y matrix of HMSC, of size *n* × *n*_*s*_; see Ovaskainen et al., 2017), we used the presence‐absence of each of the 164 MOTUs and applied a probit regression. As fixed effects (the X matrix of HMSC, of size *n* × *n*_*c*_, where *n*_*c*_ is the number of MOTU‐specific regression parameters to be estimated), we included island identity and the geographical distance to the reef of the samples. While our primary interest was in the effect of the geographical distance to the reef, we controlled for differences in geographical conditions between the islands using a transect as a random effect, thereby grouping together the two samples taken on a single transect. This random effect controlled for unexplained variation at the transect level on top of the explicitly modelled effects of distance and island identity.

We fitted the HMSC model with the R‐package *Hmsc* v.3.0.10 (Tikhonov et al., 2020), assuming the default prior distributions (see Chapter 8 of Ovaskainen & Abrego, 2020). We sampled the posterior distribution with four Markov Chain Monte Carlo (MCMC) chains, running 100,000 iterations of each chain and removing the first 50,000 as burn‐in. We thinned the chains by 100 to yield 1000 posterior samples per chain, that is 4000 posterior samples in total. We examined MCMC convergence by assessing the potential scale reduction factors (Gelman & Rubin, 1992) of the model parameters. We estimated the explanatory and predictive powers of the probit models through species‐specific values of the area under the receiver operating characteristic curve (AUC; Pearce & Ferrier, 2000) and Tjur's *R* ^2^ values (Tjur, 2009). To compute explanatory power, we made model predictions based on models fitted to all the data. To compute predictive power, we performed five‐fold cross‐validation, in which we randomly assigned the sampling units to five folds and made predictions for each fold based on a model fitted to the data on the remaining four folds. To quantify the drivers of community structure, we partitioned the explained variation among the fixed and random effects included in the model. To address our main study question, that is if and how species eDNA signals are affected by the distance to the reef, we examined species responses to the continuous explanatory variable of distance‐to‐reef. Specifically, we calculated the proportion of species that showed a positive response and the proportion that showed a negative response with at least 95% posterior probability. We multiplied all beta parameters by 100 to interpret distance‐to‐reef coefficients as the change in probability of occurrence per 100 metres. Before proceeding, we confirmed that our final models converged well, with potential scale reduction factors for the beta parameters (measuring the responses of species environmental covariates; Ovaskainen et al., 2017) of 1.001 (maximum 1.004) on average. We additionally confirmed that our model adequately fitted the data, with a mean Tjur *R* ^2^ (AUC) of .29 (0.87) for explanatory power and .17 (0.67) for predictive power.

All downstream analyses and graphics were performed in R. Data and R scripts have been deposited on the Envidat repository (https://doi.org/10.16904/envidat.497).

## 3 RESULTS

### 3.1 Overall biodiversity recovered across islands

From the total of 24,157,996 reads recovered with the species pipeline, we detected 272 different taxa (Appendix S2, Figure S2, Table S1), with 155 taxa (57.0%) assigned to the species level. When comparing the taxa list with regional faunal lists, we discarded 14 species whose spatial distribution did not match, indicating a misidentification of a closely related species not occurring in the area. We reassigned these 14 species to the genus level. We replaced one species, *Apolemichthys armitagei*, with *Apolemichthys trimaculatus*, due to its hybrid position (Heemstra & Heemstra, 2004). After these corrections, our final list of 264 detected taxa had 141 taxa (53.4%) assigned at the species level and 78 taxa (29.6%) at the genus level. In total, 14.7% (109) of the species present in local inventories were detected by eDNA. All taxonomic assignments above the genus level were discarded and not used for analysis.

The ecological and taxonomic composition of our eDNA surveys was largely consistent with local inventories. For example, of the 141 species detected, 112 (79.4%) were benthic, while 28 (19.9%) were pelagic. In comparison, local inventories assessed the presence of 743 fish species in the region, with 83.6% and 16.4% of species classified as benthic and pelagic, respectively. Across all islands, fish species recovered by eDNA mainly belonged to the Perciformes and Tetraodontiformes orders. This result was consistent across methods (67.1% Perciformes and 6.8% Tetraodontiformes for traditional surveys versus 61.1% Perciformes and 11.8% Tetraodontiformes for eDNA) and islands (Figure S2). Traditional survey methods detected a higher genus richness compared with eDNA (Figure S2; genus richness for traditional and eDNA methods, respectively: Europa = 233, 91; Glorieuse = 230, 113; Juan de Nova = 212, 44; Tromelin = 120, 42). This difference in detected genus richness was consistent with detected species richness, which was also consistently higher for traditional than for eDNA methods across all islands (Appendix S2, Figure S3; species richness for traditional and eDNA methods, respectively: Europa = 506, 131; Glorieuse = 575, 163; Juan de Nova = 477, 54; Tromelin = 238, 53). However, these differences could be partly attributed to a lack of coverage in the reference database. For example, the percentage of sequenced Perciformes presented in the TAAF faunistic list for the 12 s teleo primer is 41.7%, while it is 54.8% for the Tetraodontiformes (Appendix S2; Table S3).

Taxonomic richness detected by eDNA metabarcoding varied among the four studied islands, and the mean number of taxa detected by samples differed among islands (Kruskal–Wallis chi‐squared = 17.856, df = 3, *p*‐value <.05). Glorieuse had more taxa than both Juan de Nova (Dunn's test, stat = −3.47; *p*‐value <.05) and Tromelin (Dunn's test, stat = −3.34; *p*‐value <.05), but did not differ significantly from Europa in the number of taxa (Dunn's test, stat = 1.37; *p*‐value = 1). The eDNA surveys successfully recovered the dominance of families such as Labridae and Pomacentridae, as well as cryptic and elusive species of conservation concern. For example, Labridae was the family with the largest number of detected genera for both methods (9.0% and 15.3% of total genus richness for traditional and eDNA methods, respectively), consistently across all islands. With traditional survey methods, the five richest families (by number of detected genera) were Labridae, Gobiidae, Serranidae, Bleniidae and Pomacentridae, apart from Tromelin, where the five richest families were Labridae, Pomacentridae, Serranidae, Acanthuridae and Balistidae. For Glorieuse, the second richest family detected with eDNA was Apogonidae (6.2%), a taxon of small and cryptic species that are hard to detect and identify with traditional survey methods. Across all four islands, eDNA metabarcoding was better at detecting genera within the Myctophidae family (2.8% for eDNA versus 0.3% for traditional survey), which includes lantern and lampfish found in the bathypelagic zone during the day, such as the highseas lampfish (*Triphoturus nigrescens*). eDNA metabarcoding detected the presence of the *Oneirodes* genus, which includes deep‐water fishes, and the Aracanidae family, which includes only one species present in the WIO, namely the basketfish (*Kentrocapros rosapinto*), a deep‐water boxfish endemic to the region.

**FIGURE 2:**
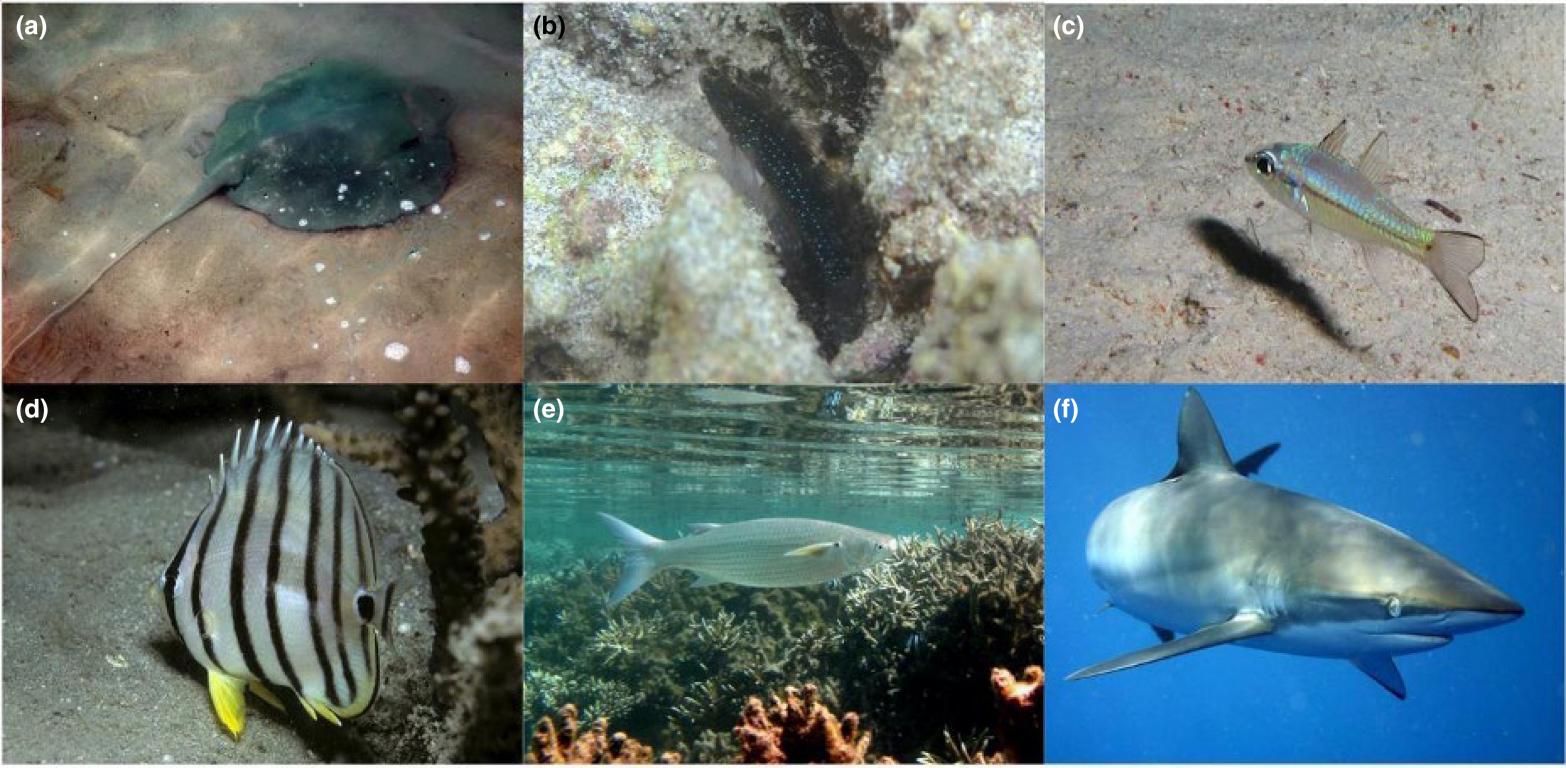
Species detected using environmental DNA that are (a–e) not listed in local inventories or (a, f) listed as ‘threatened’ in the IUCN Red List. (a) Mangrove whipray (*Urogymnus granulatus*), (b) starry goby (*Asterropteryx semipunctata*), (c) narrowstripe cardinalfish (*Pristiapogon exostigma*), (d) eight‐band butterflyfish (*Chaetodon octofasciatus*), (e) hornlip mullet (*Plicomugil labiosus*) and (f) silky shark (*Carcharhinus falciformis*). Images: Wikipedia.

Environmental DNA detected some species that were not recovered by traditional methods, such as the bony flyingfish (*Hirundichthys oxycephalus*) and the mangrove whipray (*Urogymnus granulatus*; Figure 2a), species that are rarely observed due to their elusive behaviour. Environmental DNA metabarcoding additionally detected some flounder species, such as the Indo‐Pacific oval flounder (*Bothus myriaster*). Moreover, eDNA metabarcoding detected cryptic species difficult to detect by divers, either because they hide during the day or because they are small, such as the starry goby (*Asterropteryx semipunctata*; Figure 2b) and the narrowstripe cardinalfish (*Pristiapogon exostigma*; Figure 2c). Environmental DNA metabarcoding detected Schindler's fish (*Schindleria praematura*), a goby that manifests retention of juvenile characteristics (paedomorphism). Additionally, this method detected seven species that are listed as ‘threatened – vulnerable’ in the IUCN Red List: the silky shark (*Carcharhinus falciformis*; Figure 2f), the tawny nurse shark (*Nebrius ferrugineus*), the honeycomb stingray (*Himantura uarnak*), the Chilean devil ray (*Mobula tarapacana*), the porcupine ray (*Urogymnus asperrimus*), the mangrove whipray (*Urogymnus granulatus*; Figure 2a) and the brown‐marbled grouper (*Epinephelus fuscoguttatus*). Environmental DNA metabarcoding also detected the blue‐spotted stingray (*Neotrygon kuhlii*), which is listed as ‘data deficient’ in the IUCN Red List.

### 3.2 Environmental DNA MOTU composition among islands

With the MOTU pipeline, we detected 356 MOTUs for a total of 32,407,191 reads (Appendix S2, Table S2). On average, we identified 61.4 ± 7.3 MOTUs (as a proxy for species) per sample. Moreover, recovered MOTU richness varied among the four studied islands, and the mean number of MOTUs detected by samples differed among islands (Kruskal–Wallis chi‐squared = 16.672, df = 3, *p*‐value = .0008253). Glorieuse had a larger number of MOTUs on average than both Juan de Nova (Dunn's test, stat = −3.56; *p*‐value = .0023) and Tromelin (Dunn's test, stat = −3.06; *p*‐value = .013), but did not differ significantly from Europa in the number of MOTUs (Dunn's test, stat = 1.49; *p*‐value = .82). The total number of MOTUs identified was 235 for Europa, 265 for Glorieuse, 93 for Juan de Nova and 112 for Tromelin.

MOTU composition dissimilarity varied among the eDNA samples collected on the four islands. The first two axes of the PCoA explained 36.6% of the total dataset inertia, with 24.4% for the first axis and 12.2% for the second axis (Figure 1f). Differences in MOTU composition were particularly marked between Europa and Glorieuse, while MOTU composition was similar for Juan de Nova and Tromelin.

### 3.3 Compositional changes in response to distance from the reef

**FIGURE 3:**
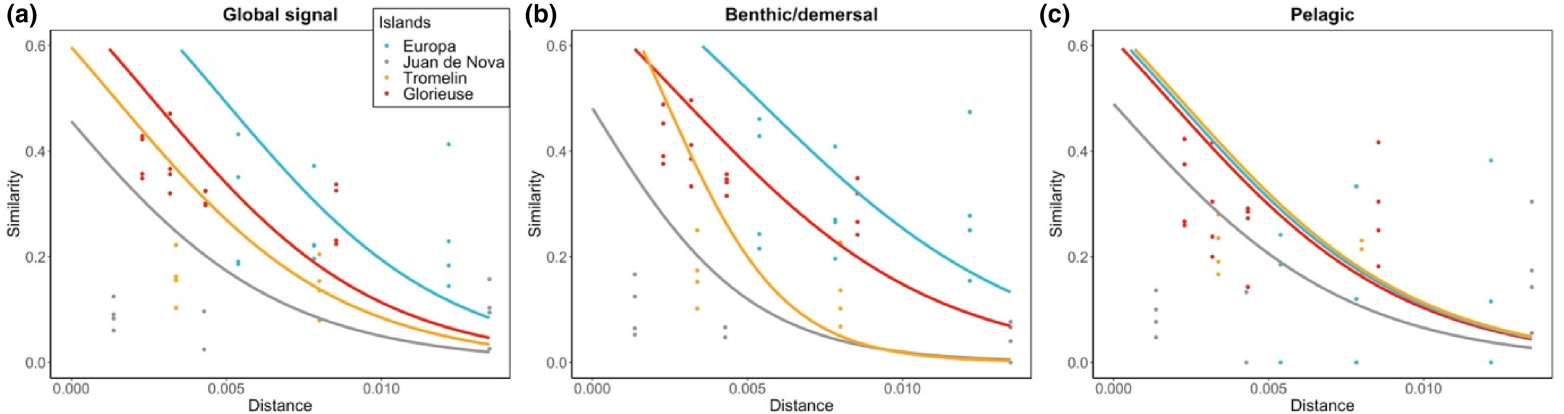
Relationship between molecular operational taxonomic unit (MOTU) compositional similarity (Jaccard index) and spatial distance (in degree) between pairs of samples, with one sample taken directly over the reef and the second taken away from the reef. (a) Relationship for all MOTUs for each island (Europa, Juan de Nova, Tromelin and Glorieuse). (b) Relationship for benthic MOTUs and (c) Relationship for pelagic MOTUs. Each point represents the species similarity between a pair of samples. The lines represent the fit of a GLM model, accounting for the distance and the interaction between island and distance.

MOTU composition shifted with the eDNA signal with increasing distance from the reefs. Samples taken at increasing distances from the reefs were significantly more different than those taken in proximity to them (GLMM, *p* = .016; Figure 3a; Appendix S3, Table S4) in a mixed effects model accounting for the pairs of samples per transect. This shift in MOTU composition with increasing distance from the reefs was also consistent when examining species associated with benthic (GLMM, *p* = .022; Figure 3b,c; Table S4) and pelagic habitats (GLMM, *p* = .014; Figure 3b,c; Table S4), with no marked differences in the responses of these two groups. We found no significant interaction effect with island identities for the three models, indicating that the change in species composition followed the same trend at all locations (GLMM, all *p* > .05; Figure 3a; Table S4).

### 3.4 MOTU response to covariates

**FIGURE 4:**
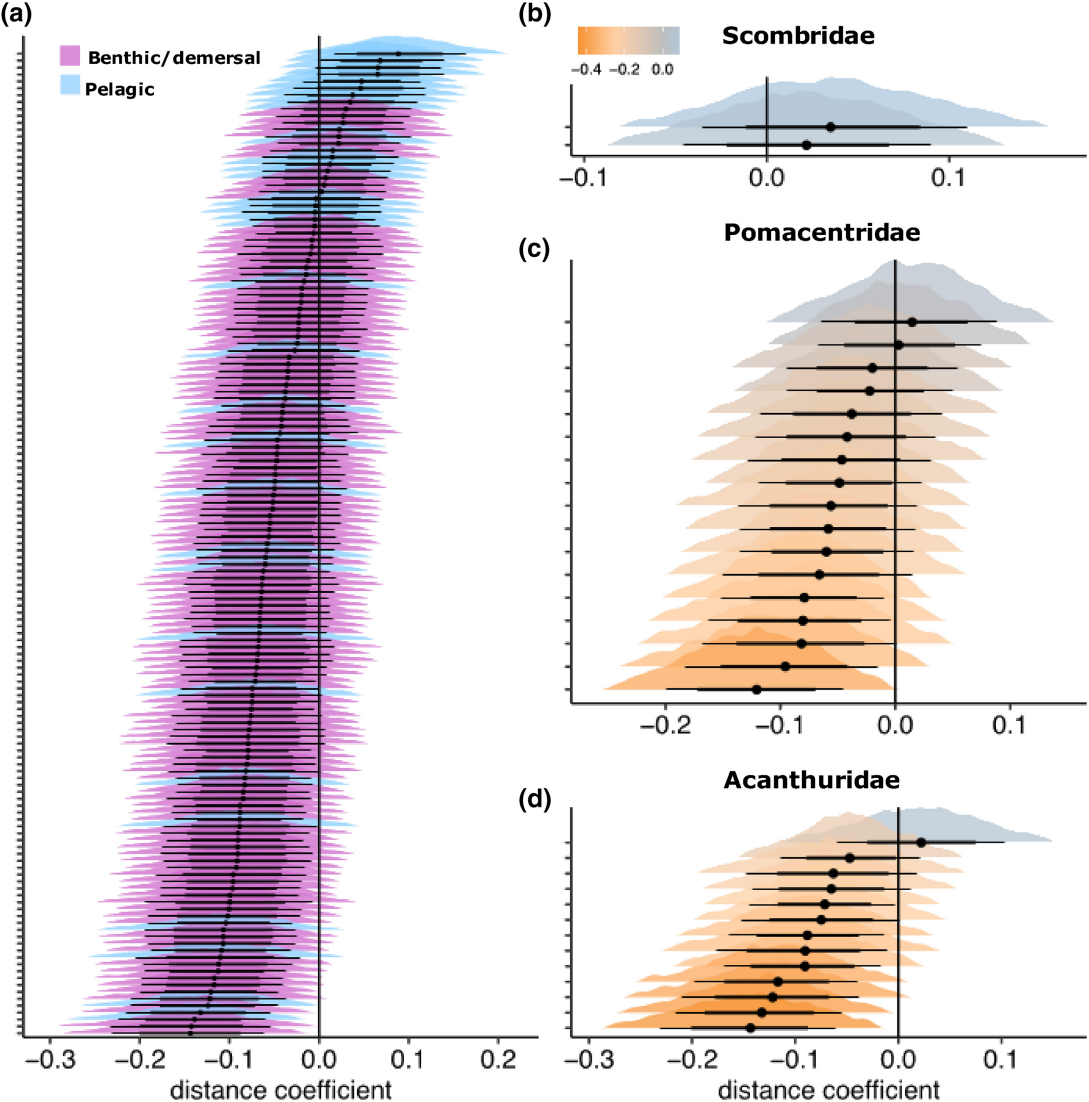
Effect of distance to the reef on the occurrence probability of molecular operational taxonomic units (MOTUs) detected by environmental DNA. We modelled MOTU occurrence probability using a hierarchical modelling of species communities (HMSC) statistical framework, which enabled us to estimate a separate distance coefficient for each MOTU. (a) overall effect of distance for 143 MOTUs, (b) a subset of pelagic MOTUs from the Scombridae family; (c) mixed‐habitat MOTUs from the Pomacentridae family and (d) benthic MOTUs from the Acanthuridae family. In (a), species coefficient estimates are coloured by pelagic (blue) and benthic habitat type (purple), whereas in (b–d) colour represents the effect of distance. We calculated 4000 posterior estimates for each MOTU. Each horizontal line represents a MOTU, with coloured histograms showing the 95% confidence intervals of the parameter posterior estimates. Points represent the median of the posterior estimates, and thick solid lines and thin solid lines represent the 60% and 80% confidence intervals, respectively. The vertical line in each panel represents the reference point used to determine the coefficient's significance.

We found a MOTU‐specific response to the distance from the reef, with some MOTUs generally occurring near the reef and others occupying the pelagic environment farther from the reef. In general, benthic MOTUs were detected closer to the reef (Figure 4a,c,d; coefficient below the vertical line), while typical pelagic MOTUs were found at greater distances from the reef, regardless of the island in question (Figure 4b; coefficient above the vertical line). Moreover, in our model island identify explained 24.4% of the total model variance, distance to the reef explained 2.6% and the random effect of transect explained 1.8% (Appendix S4, Figure S4a). When considering the explained variance only, islands explained 81.8% of the explained variation in the model, distance to the reef explained 10.5% and within‐transect replicates explained 7.7% (Figure S4b). We estimated a separate distance coefficient for each of the 143 MOTUs (Figure 4; Appendix S4, Table S5).

## 4 DISCUSSION

Environmental DNA metabarcoding has been increasingly used to investigate marine biodiversity in the past decade, yet the diffusion of eDNA and the biodiversity signals recovered from eDNA at high resolution remain overlooked (West et al., 2020). Here, we show a marked turnover in composition between eDNA samples taken on reefs and those taken hundreds of metres from the reefs. Moreover, by coupling fine‐scale inventories of eDNA with JSDMs, we provide evidence for a fine‐scale distribution of eDNA biodiversity signals. At a larger scale, our data reveals major compositional differences in biodiversity between remote islands, highlighting the ability of eDNA to distinguish biogeographical patterns and complement fish species inventories for remote islands (Mathon et al., 2022; Polanco Fernández, Marques, et al., 2021). As a non‐invasive, cost‐ and time‐efficient technique, eDNA is well suited to advancing biodiversity monitoring in oceans, which is one of the goals set by UNESCO's Decade of Ocean Science for Sustainable Development (2021–2030; UNESCO‐IOC, 2021).

Traditional surveys of species occurrences along fine‐scale environmental gradients can be complemented by data derived from eDNA (Jeunen et al., 2019). We observed a marked compositional turnover of MOTUs along a gradient of distance from the reef (Figure 3). We further combined eDNA metabarcoding with JSDMs to detect species‐level occupancy along a distance to reef gradient across the four considered islands (Figure 4). Our results suggest that eDNA metabarcoding can be used to identify an ecological signal of habitat selection by fish species and MOTUs across the transition from coral reefs to pelagic habitat. Rather than suggesting a broad diffusion of eDNA, our approach indicates that eDNA is detected only over small spatial distances from where it originated, as already demonstrated in numerous studies (Lafferty et al., 2021; Murakami et al., 2019; Nguyen et al., 2020; Polanco Fernández, Marques, et al., 2021; Rozanski et al., 2022). As expected, we detected species belonging to the Scombridae family with greater probability in their pelagic habitat (Colette & Nauen, 1983). Likewise, we observed an eDNA signal of a shark species (Carcharhinidae family) close to coral reefs. In the same way, the eye‐bar goby (*Gnatholepis anjerensis*) and other typical reef‐associated species within the Acanthuridae family had higher occurrence probabilities far from the reef. This finding suggests a signal of the diurnal spatial use of coral reefs by typical benthic species (Hitt et al., 2011) or the detection of pelagic larvae (Leis & McCormick, 2002; Rocha et al., 2002), as eDNA metabarcoding is not yet capable of life stage delimitation (Beng & Corlett, 2020). Nevertheless, we detected taxa belonging to deep‐water species such as Myctophidae that were not detected by typical inventory methods. These detections are not contradictory to our fine‐scale environmental gradients because these taxa likely originate from nearby waters due to their diel vertical migration during the night for feeding (Mathon et al., 2022; Watanabe et al., 2002). Furthermore, these islands feature steep drop‐offs, leading mesopelagic species to migrate to shallow waters during the night, near the islands. Finally, our results illustrate the potential for eDNA metabarcoding to disentangle the spatial occupancy of fish species despite confounding factors that mix ocean environments, suggesting that eDNA could be used to infer the occupancy and use of space by species on coral reefs.

When combining statistical approaches based on community data (such as JSDMs, implemented here using HMSC) with eDNA metabarcoding, data on species identity and the unique ecology of species can be retained, information that is ignored when aggregating the data into overall species/MOTU richness (Ovaskainen & Abrego, 2020). Therefore, community modelling approaches could make better use of valuable information contained in eDNA‐based biodiversity data to reveal clearer signals of reef spatial occupancy by marine fishes. Conversely, when generating large spatial community data, eDNA metabarcoding could greatly benefit from the combination with JSDMs (Ovaskainen et al., 2017; Pichler & Hartig, 2020), such as the novel HMSC framework. HMSC models the responses of rare species and their potentially unique responses, characterises species occurrences in relation to environmental attributes, and has the capability to identify community assemblage processes (Ovaskainen & Abrego, 2020). Until recently, one challenge in applying such statistical tools was how to scale them computationally to massive data sets (Warton et al., 2015), such as those produced by eDNA. By using latent variables to replace heavy spatial covariance matrices, modern HMSC makes it possible to analyse large spatio‐temporal data generated through high‐throughput molecular technologies (Pichler & Hartig, 2020; Tikhonov et al., 2020). By enhancing the information content recovered from eDNA‐based data and by applying HMSC, we are likely to better inform decision‐making actions in ecosystem conservation and management (Burian et al., 2021).

Large‐scale biogeographical patterns across islands can be revealed by eDNA metabarcoding. Using sequence clustering to delineate MOTUs, in our study we identified differences in community composition across remote islands (Polanco Fernández, Marques, et al., 2021). Our findings highlight inter‐island differences in benthic community richness but do not indicate differences in pelagic MOTU richness across the islands. The diversity of some typical pelagic species might be underestimated due to the lack of variation in the ‘teleo’ primer employed here for some taxa, such as *Thunnus* and *Scarus* (Polanco Fernández, Richards, et al., 2021). However, pelagic communities are expected to differ less between islands than benthic assemblages, as most pelagic species rely on coral reefs for food provisioning, spawning or nursery ground but can travel far distances (Chin et al., 2013; McCauley et al., 2012, 2016), as reflected in our eDNA‐based survey. Inter‐island variation highlights differences not only in island marine fish composition but also in eDNA detection according to hydrological parameters. We detected marked differences in the eDNA signals across islands, which might be due to differences in sea conditions (Barnes & Turner, 2015; Stewart, 2019) and seascapes (Nguyen et al., 2020). For example, the species compositions in Juan De Nova and Tromelin were more similar than in the two other islands, probably because of their lower species richness and their reef condition. While Juan de Nova has a relatively large reef area, we sampled there just after a hurricane, which could have caused its species composition to be more like that in the isolated Tromelin, which has low coral coverage. Therefore, future marine eDNA study designs might benefit from the integration of hydrological, geomorphological and abiotic parameters differentiating coastal areas (Carraro et al., 2020, 2021; Pilliod et al., 2014).

Our results highlight the capability of eDNA to provide information to potentially enrich island faunal lists performed with traditional survey methods on isolated coral reefs. However, many species present in the islands' faunal lists were not detected in the eDNA surveys, a result with several possible reasons. First, given that only 25% of the species in the WIO are sequenced for the ‘teleo’ primer, many species are likely to have been missed by eDNA due to their absence in the reference databases, reflecting the need for the continual development of the database (Marques et al., 2021). To diminish this drawback and obtain an overview of the overall biodiversity, we used sequence clustering with SWARM to generate MOTUs as a proxy for species even without a complete reference database (Marques et al., 2020), which increased the detected taxa richness by a factor of 2.5 (141 species versus 356 MOTUs). Similarly, by constructing an additional reference database of 67 species, Valdivia‐Carrillo et al. (2021) tripled the taxonomic assignment of reads (see also Sales et al., 2021). Second, the sampling effort for traditional surveys exceeded that of eDNA (23 years of sampling compared with a single time‐point), and a larger number of samples could have led to a broader coverage of species (Valdivia‐Carrillo et al., 2021). Nonetheless, the Scattered Islands data sets generated from eDNA‐based surveys are likely to characterise changes in coral reef community assemblages (West et al., 2020) and enhance our ability to monitor coral reef ecosystems globally (Mathon et al., 2022).

Given the growing anthropogenic pressure on coral reefs and the alarming rates of biodiversity decline, marine ecosystems are in urgent need of efficient and reliable monitoring programmes to protect their magnificent biodiversity and identify zones of conservation priority (O'Hara et al., 2021; Sala et al., 2021). We show that eDNA metabarcoding enables surveys of species occupancy along fine‐scale distance gradients associated with contrasting environmental conditions. Further, our identification of fine‐scale spatial structure from eDNA highlights applications of this metabarcoding technique in providing temporally resolved data for responsive monitoring programmes that need to quickly address problems and set conservation plans for our changing oceans (e.g., in response to extreme climate events; Berry et al., 2019). The continuous development of eDNA metabarcoding applications to detect biodiversity and the upscaling of their use globally with standardised protocols will enhance the establishment of conservation priorities and management plans in remote regions (Boussarie et al., 2018; Marques et al., 2020; Ruppert et al., 2019).

## AUTHOR CONTRIBUTIONS

**Mélissa Jaquier:** Data curation (equal); formal analysis (equal); writing – original draft (equal). **Camille Albouy:** Conceptualization (equal); formal analysis (equal); methodology (equal); project administration (lead); resources (equal); supervision (equal); visualization (equal); writing – original draft (equal); writing – review and editing (equal). **Wilhelmine Bach:** Writing – original draft (equal). **Conor Waldock:** Data curation (equal); formal analysis (equal); visualization (equal); writing – review and editing (equal). **Virginie Marques:** Data curation (equal); investigation (equal); methodology (equal); resources (equal); writing – review and editing (equal). **Eva Maire:** Methodology (equal); project administration (equal); writing – review and editing (equal). **Jean Baptiste Juhel:** Investigation (equal); writing – review and editing (equal). **Marco Andrello:** Investigation (equal); project administration (supporting); writing – review and editing (equal). **Alice Valentini:** Data curation (equal); writing – review and editing (equal). **Stéphanie Manel:** Conceptualization (equal); funding acquisition (equal); project administration (lead); writing – review and editing (equal). **Tony Dejean:** Funding acquisition (supporting); validation (supporting); writing – review and editing (supporting). **David Mouillot:** Conceptualization (equal); project administration (lead); supervision (equal); validation (equal); writing – review and editing (equal). **Loïc Pellissier:** Conceptualization (lead); funding acquisition (lead); methodology (equal); project administration (supporting); supervision (lead); validation (lead); writing – original draft (equal); writing – review and editing (equal).

## CONFLICT OF INTEREST STATEMENT

The authors declare there are no conflicts of interests.

## Supporting information


Appendices S1–S4.

## Data Availability

The R‐scripts and the raw sequencing data (https://www.doi.org/10.16904/envidat.497) are archived online and are publicly available.
