# Supplementary material for: Environmental DNA recovers fish composition turnover of the coral reefs of West Indian Ocean islands
Source: Ecol Evol. 2024 May 16;14(5):e11337. doi: 10.1002/ece3.11337 (PMC11099785; doi:10.1002/ece3.11337)
Supplement: Supplementary file 1 — Appendices S1–S4. [file ECE3-14-e11337-s001.docx]

Supplementary Material

**eDNA recovers fish composition turnover of the coral reefs of West Indian Ocean islands**

# **Supplementary Material 1: Detailed materials and methods of eDNA laboratory protocol and bioinformatic pipelines**

eDNA laboratory protocol

Following the protocol of Polanco Fernández et al. (2021), we performed DNA extraction twice per filtration capsule and then pooled the two extracted DNA samples prior to the amplification procedure. Following the protocol of Biggs et al. (2015), we tested samples for inhibition by qPCR after extraction. If a sample was considered inhibited, we diluted it five-fold prior to amplification. We used one primer for amplification, targeting teleosteans (teleo), a 12s primer selecting for Actinopterygii and Elasmobranchii (Valentini et al., 2016). We carried out DNA amplifications in a final volume of 25 µL, using 3 µL of DNA extract as a template. The amplification mixture consisted of 1 U AmpliTaq Gold DNA polymerase (Applied Biosystems, Foster City, CA, USA), 10 mM Tris-HCL, 50 mM KCL, 2.5 mM MgCl2, 0.2 mM each dNTP, 0.2 µM teleo primers, 4 µM human blocking primer for the teleo primers (Civade et al., 2016), and 0.2 µg/µL bovine serum albumin (BSA; Roche Diagnostics, Basel, Switzerland). We 5’-labelled the teleo primer pair with an eight-nucleotide tag exclusive to each PCR replicate, with at least three dissimilarities between any pair of tags. This allowed the allocation of each sequence to the corresponding sample during sequence analysis. For each PCR replicate, we used identical tags for the forward and reverse primers. For each filter, we ran 12 replicate PCRs to amplified DNA in a dedicated room with negative air pressure, to physically separate this step from DNA extraction, which we performed in rooms with positive air pressure. We denatured the PCR mixture at 95°C for 10 min, followed by 50 cycles of 30 s at 95°C, 30 s at 55°C, and 1 min at 72°C, and a final elongation step at 72°C for 7 min. After amplification, we titrated the samples using capillary electrophoresis (QIAxcel; Qiagen GmbH, Hilden, Germany) and purified them using the MinElute PCR purification kit (Qiagen GmbH). We titrated the purified DNA, again with capillary electrophoresis, prior to sequencing. Finally, we merged the purified PCR outputs into equal volumes to achieve a theoretical sequencing depth of 500,000 reads per sample. Library preparation and sequencing steps were completed at Fasteris (Geneva, Switzerland). One library was assembled using the MetaFast protocol (Fasteris 2020).

Comparison of eDNA taxon assignments with local inventories

Incomplete and erroneous genetic reference databases are two of the main limitations of the eDNA approach, biasing sequence assignment to taxonomic levels. To additionally restrict the eDNA taxonomic assignments, we verified the recovered taxonomic assignments from eDNA with local faunal lists (comm. pers. Terres Australes et Antarctiques Françaises, TAAF) when possible, or using the Fishbase database (Froese & Pauly, 2021). For both bioinformatic pipelines (species and MOTU), we paired eDNA records with lists of regional species pools and validated the species, genus or family captured by eDNA that has been reported to be present in the area. We upped the taxonomic level of taxa that are not present in the WIO. We discarded species, genera or families known to live in freshwater or representing known lab contaminants (e.g., *Salmo salar*). We reassigned detected species, genera or families known to occur only in other areas to the immediate higher taxon known to occur in the region (e.g., *Canthigaster jactator*, known to be endemic to Hawaii, was reassigned to the *Canthigaster* genus).


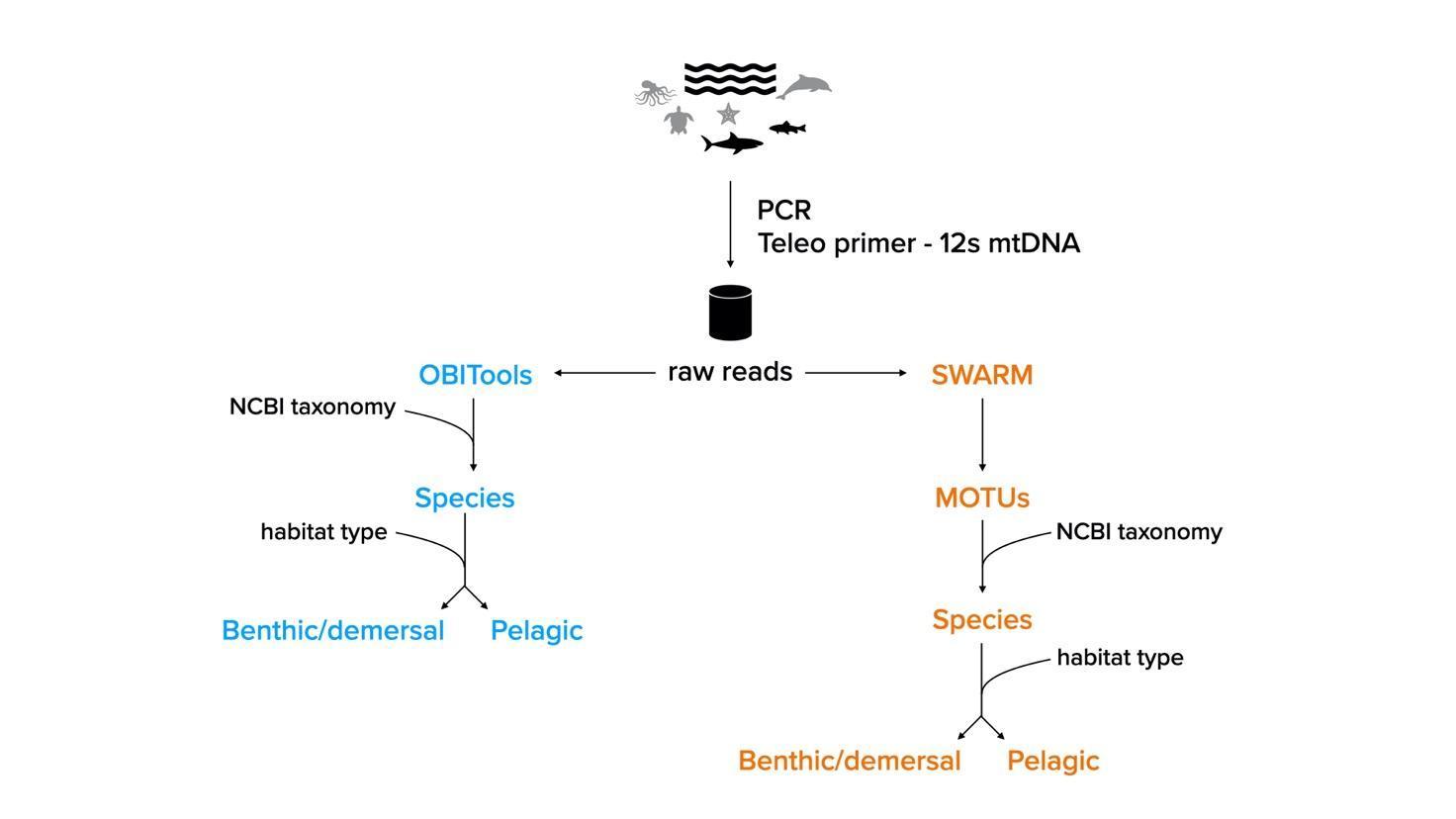


**Figure S1:** Conceptual diagram illustrating how we used two bioinformatic pipelines to generate lists of species and MOTUs detected within each filter in our environmental DNA (eDNA) metabarcoding study. From eDNA extracted by filtering 30 L of seawater, we selected Actinopterygii and Elasmobranchii by amplification through polymerase chain reaction (PCR), with the teleo/Tele01 primer. We then analysed DNA sequences through species (blue) and MOTU (orange) bioinformatic pipelines. In the species pipeline, we compared sequences identified with the OBITools toolkit with the NCBI taxonomic reference database to generate species lists. In the MOTU pipeline, we used a SWARM algorithm to cluster sequences into MOTUs, which we then compared with the NCBI database to generate species lists. In both pipelines, we classified species into the two habitat types benthic and pelagic, according to information found in the Fishbase database.

References

Biggs, J., Ewald, N., Valentini, A., Gaboriaud, C., Dejean, T., Griffiths, R. A., Foster, J., Wilkinson, J. W., Arnell, A., Brotherton, P., Williams, P., & Dunn, F. (2015). Using eDNA to develop a national citizen science-based monitoring programme for the great crested newt (*Triturus cristatus*). Biological Conservation, *183*, 19–28. <https://doi.org/10.1016/J.BIOCON.2014.11.029>

Civade, R., Dejean, T., Valentini, A., Roset, N., Raymond, J. C., Bonin, A., Taberlet, P., & Pont, D. (2016). Spatial representativeness of environmental DNA metabarcoding signal for fish biodiversity assessment in a natural freshwater system. PLoS ONE, 11(6), e0157366. <https://doi.org/10.1371/JOURNAL.PONE.0157366>

Fasteris. 2020. MetaFast Protocol for PCR Amplicon Analysis of Metagenomic DNA. Retrieved from https://www.fasteris.com/dna/?q=content/metafast-protocol-amplicon-metagenomic-analysis

Froese, R. & Pauly, D. (eds) (2021) FishBase. World WideWeb electronic publication. Version 03/2021. Available at: http//www.ﬁshbase.org

Polanco Fernández, A., Marques, V., Fopp, F., Juhel, J. B., Borrero-Pérez, G. H., Cheutin, M. C., Dejean, T., González Corredor, J. D., Acosta-Chaparro, A., Hocdé, R., Eme, D., Maire, E., Spescha, M., Valentini, A., Manel, S., Mouillot, D., Albouy, C., & Pellissier, L. (2021). Comparing environmental DNA metabarcoding and underwater visual census to monitor tropical reef fishes. Environmental DNA, 3(1), 142–156. <https://doi.org/10.1002/EDN3.140>

Valentini, A., Taberlet, P., Miaud, C., Civade, R., Herder, J., Thomsen, P. F., Bellemain, E., Besnard, A., Coissac, E., Boyer, F., Gaboriaud, C., Jean, P., Poulet, N., Roset, N., Copp, G. H., Geniez, P., Pont, D., Argillier, C., Baudoin, J. M., … Dejean, T. (2016). Next-generation monitoring of aquatic biodiversity using environmental DNA metabarcoding. Molecular Ecology, 25(4), 929–942. https://doi.org/10.1111/MEC.13428

#
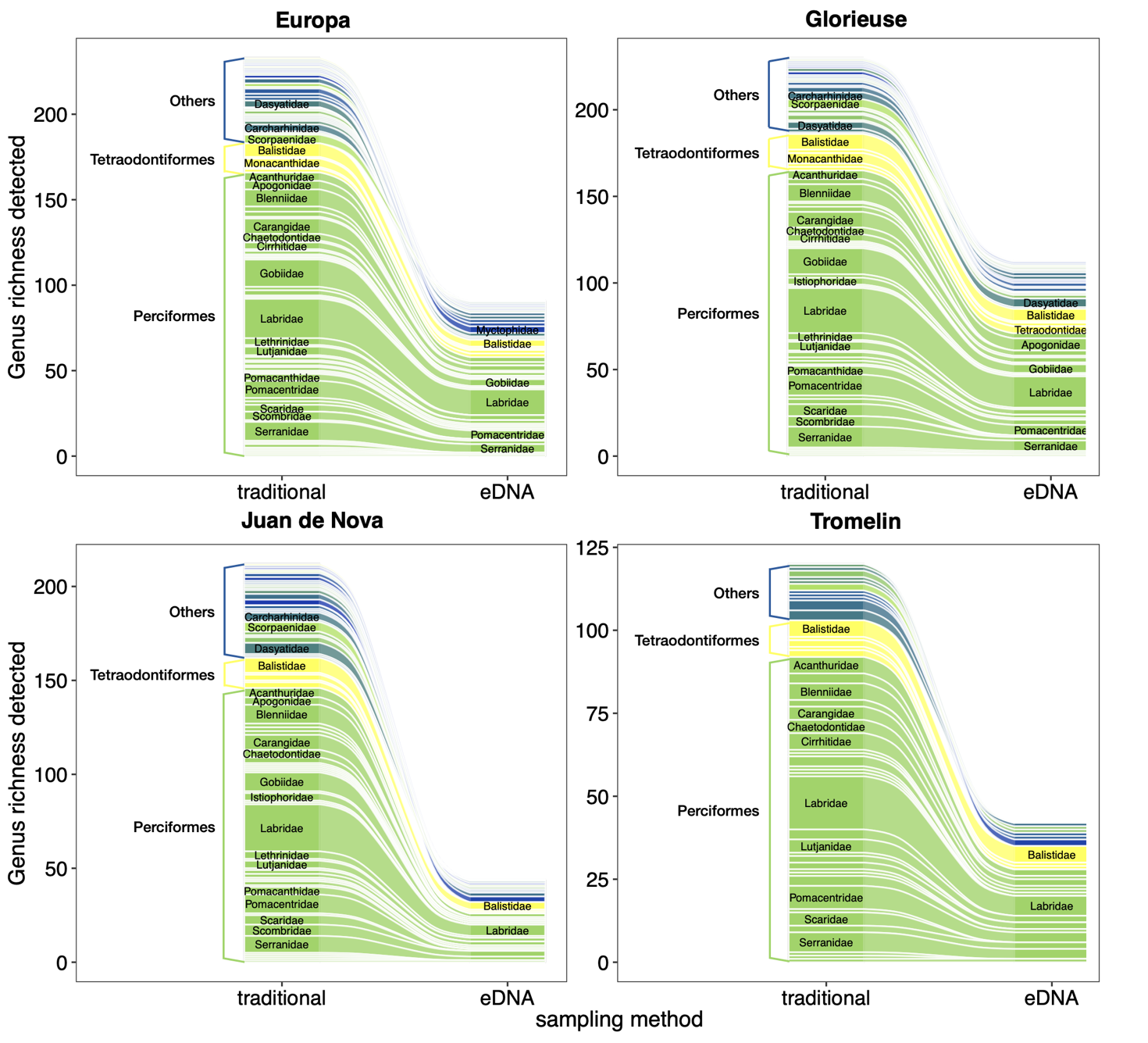
 **Supplementary Material 2: Comparison of eDNA and traditional survey methods and taxa detected by both bioinformatic pipelines.**

**Figure S2:** Sankey diagrams showing the detection capabilities of eDNA (one sampling event) compared with traditional methods (23 years of surveys) across the four studied islands in the French Scattered Islands. The x-axis shows the two different sampling methods, while the y-axis shows the genus richness detected for the island and for each family. Perciformes are represented in green, Tetraodontiformes in yellow, and other orders families in blue.


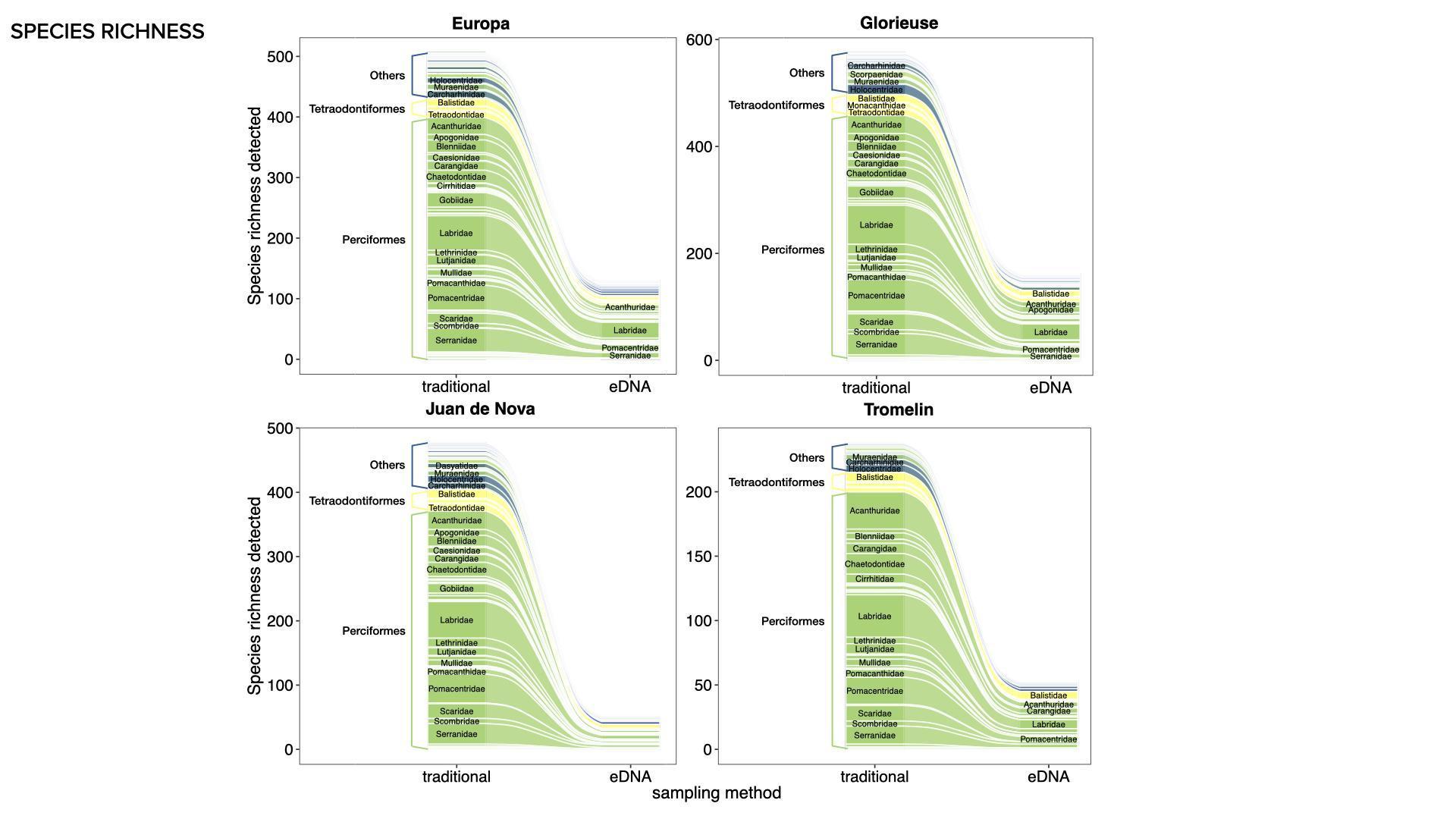


**Figure S3:** Sankey diagrams showing the species detection capabilities of eDNA (one sampling event) compared with traditional methods (23 years of surveys) across the four studied islands in the French Scattered Islands. The x-axis shows the two different sampling methods, while the y-axis shows the species richness detected for the island and for each family. Perciformes are represented in green, Tetraodontiformes in yellow, and other families in blue.

**Table S1:** Number of sequences detected per taxa using the “species pipeline” at the site levels.

|  | europa_ 0m | europa_ 300m | europa _50m | europa_ 600m | glorieuse | glorieuse _0m | glorieuse_100m | glorieuse_300m | glorieuse _50m | juan_0 | juan_1 | Juan_2 | Juan_3 | taaf_station | taaf_station2 | tromelin _0m | tromelin_ 100m | tromelin_ 50m |
| --- | --- | --- | --- | --- | --- | --- | --- | --- | --- | --- | --- | --- | --- | --- | --- | --- | --- | --- |
| Abudefduf | 299820 | 0 | 15858 | 15807 | 50917 | 252703 | 18670 | 0 | 131026 | 0 | 0 | 0 | 0 | 135844 | 850 | 592 | 0 | 0 |
| Abudefduf notatus | 216 | 0 | 2330 | 673 | 0 | 0 | 0 | 0 | 0 | 0 | 0 | 0 | 0 | 0 | 0 | 0 | 0 | 0 |
| Abudefduf septemfasciatus | 1916 | 4254 | 757 | 0 | 0 | 0 | 0 | 0 | 18518 | 0 | 0 | 0 | 0 | 0 | 5559 | 0 | 0 | 0 |
| Acanthuridae | 170667 | 42716 | 88243 | 118493 | 617645 | 321422 | 96488 | 30919 | 237654 | 48562 | 198954 | 0 | 0 | 430938 | 75087 | 10713 | 0 | 15225 |
| Acanthurus | 942718 | 794944 | 2406437 | 223695 | 208629 | 328242 | 225732 | 11699 | 343809 | 133 | 0 | 5895 | 0 | 408338 | 738770 | 101349 | 0 | 0 |
| Acanthurus bariene | 0 | 0 | 0 | 0 | 0 | 0 | 0 | 0 | 843 | 0 | 0 | 0 | 0 | 0 | 0 | 0 | 0 | 0 |
| Acanthurus lineatus | 114851 | 13660 | 90588 | 32124 | 317614 | 178376 | 23914 | 4783 | 25405 | 2033 | 554121 | 0 | 0 | 0 | 44132 | 0 | 0 | 0 |
| Acanthurus nigrofuscus | 272625 | 209356 | 294054 | 1457 | 416944 | 83988 | 277708 | 0 | 202284 | 42 | 25136 | 0 | 1108 | 17 | 456 | 95232 | 0 | 35 |
| Aethaloperca rogaa | 299 | 0 | 0 | 0 | 0 | 0 | 15388 | 0 | 2943 | 0 | 99162 | 0 | 2386 | 0 | 3386 | 198985 | 47786 | 0 |
| Albula | 185 | 0 | 0 | 0 | 0 | 21754 | 3572 | 0 | 27802 | 0 | 0 | 0 | 0 | 0 | 0 | 0 | 0 | 0 |
| Alepocephaliformes | 0 | 0 | 0 | 0 | 7599 | 0 | 0 | 0 | 0 | 0 | 0 | 0 | 0 | 0 | 0 | 73073 | 8873 | 0 |
| Amphiprion akallopisos | 0 | 0 | 0 | 0 | 12 | 167 | 0 | 0 | 0 | 0 | 0 | 0 | 0 | 0 | 0 | 0 | 0 | 0 |
| Anampses | 0 | 0 | 0 | 0 | 71 | 3066 | 0 | 0 | 2580 | 0 | 0 | 0 | 0 | 0 | 0 | 0 | 0 | 0 |
| Anguilliformes | 1284 | 17617 | 0 | 0 | 7157 | 1557 | 32 | 1973 | 0 | 0 | 0 | 0 | 0 | 0 | 5432 | 0 | 0 | 0 |
| Aphaniidae | 0 | 0 | 0 | 0 | 0 | 0 | 0 | 0 | 0 | 0 | 0 | 0 | 0 | 0 | 0 | 64840 | 0 | 0 |
| Apogon semiornatus | 0 | 0 | 0 | 0 | 21128 | 35 | 0 | 0 | 419 | 0 | 0 | 0 | 0 | 0 | 0 | 0 | 0 | 0 |
| Apolemichthys armitagei | 0 | 0 | 0 | 0 | 0 | 0 | 0 | 1730 | 0 | 0 | 0 | 0 | 0 | 0 | 0 | 0 | 0 | 0 |
| Ariosoma | 4920 | 0 | 0 | 0 | 0 | 0 | 0 | 0 | 0 | 0 | 0 | 0 | 0 | 0 | 0 | 0 | 0 | 0 |
| Arothron | 321 | 357562 | 0 | 0 | 10214 | 13572 | 83788 | 90722 | 13160 | 0 | 0 | 0 | 0 | 12523 | 0 | 0 | 0 | 0 |
| Arothron mappa | 0 | 0 | 0 | 0 | 6575 | 0 | 0 | 0 | 1537 | 0 | 0 | 0 | 0 | 0 | 0 | 0 | 0 | 0 |
| Asterropteryx | 0 | 0 | 0 | 0 | 0 | 0 | 12552 | 0 | 0 | 0 | 0 | 0 | 0 | 0 | 0 | 0 | 0 | 0 |
| Atherinidae | 45624 | 83424 | 7190 | 0 | 7750 | 42102 | 3940 | 0 | 1260 | 0 | 0 | 0 | 0 | 0 | 0 | 0 | 0 | 0 |
| Atherinomorphae | 0 | 0 | 0 | 0 | 636408 | 57127 | 22896 | 1532 | 47382 | 0 | 0 | 0 | 0 | 29 | 0 | 174 | 0 | 0 |
| Auxis | 0 | 0 | 0 | 0 | 0 | 0 | 0 | 0 | 0 | 570 | 0 | 0 | 15862 | 0 | 0 | 0 | 0 | 0 |
| Balistapus undulatus | 1095 | 648 | 86791 | 6454 | 84869 | 4502 | 14751 | 0 | 12924 | 0 | 40398 | 0 | 0 | 0 | 0 | 0 | 0 | 0 |
| Balistoides conspicillum | 0 | 0 | 0 | 0 | 0 | 0 | 0 | 0 | 688 | 0 | 0 | 0 | 0 | 0 | 0 | 555 | 0 | 0 |
| Belonidae | 188556 | 13456 | 6810 | 2566 | 3321 | 1352 | 0 | 0 | 0 | 0 | 0 | 0 | 0 | 0 | 92125 | 113926 | 0 | 0 |
| Benthosema | 250 | 0 | 0 | 0 | 952 | 107 | 24949 | 9374 | 11368 | 0 | 0 | 0 | 0 | 0 | 0 | 0 | 0 | 0 |
| Blenniiformes | 0 | 0 | 0 | 4730 | 0 | 0 | 0 | 0 | 0 | 255731 | 0 | 0 | 0 | 0 | 0 | 0 | 0 | 0 |
| Bolinichthys | 2964 | 0 | 0 | 0 | 73466 | 0 | 0 | 4251 | 0 | 37703 | 1129 | 36196 | 9312 | 0 | 408 | 46177 | 0 | 915 |
| Caesio | 1286 | 12401 | 3676 | 348 | 96289 | 36403 | 13947 | 24465 | 23560 | 0 | 6636 | 0 | 0 | 27672 | 23717 | 13689 | 0 | 0 |
| Caesio teres | 11284 | 40548 | 862 | 37728 | 281349 | 45018 | 133862 | 50151 | 5523 | 639648 | 72112 | 0 | 0 | 717 | 14688 | 0 | 0 | 0 |
| Cantherhines pardalis | 0 | 11649 | 0 | 0 | 0 | 0 | 0 | 0 | 2752 | 0 | 100885 | 24354 | 1270 | 0 | 0 | 4788 | 0 | 0 |
| Canthidermis maculata | 0 | 0 | 0 | 0 | 0 | 0 | 0 | 0 | 5297 | 13 | 0 | 0 | 0 | 0 | 0 | 0 | 0 | 0 |
| Canthigaster | 0 | 0 | 576 | 1978 | 0 | 15277 | 0 | 18423 | 6962 | 0 | 0 | 0 | 0 | 0 | 0 | 0 | 0 | 0 |
| Canthigaster valentini | 0 | 0 | 2005 | 0 | 0 | 0 | 0 | 0 | 0 | 0 | 0 | 0 | 0 | 0 | 0 | 0 | 0 | 0 |
| Carangidae | 0 | 0 | 0 | 0 | 0 | 24581 | 0 | 0 | 19 | 0 | 0 | 0 | 0 | 0 | 0 | 0 | 0 | 0 |
| Caranx | 0 | 0 | 0 | 0 | 0 | 0 | 0 | 0 | 0 | 0 | 0 | 0 | 0 | 0 | 0 | 0 | 3581 | 0 |
| Caranx melampygus | 342 | 0 | 0 | 0 | 1675 | 1511 | 52585 | 347 | 5286 | 0 | 0 | 0 | 0 | 52 | 0 | 4164 | 12 | 3107 |
| Carcharhinidae | 30 | 0 | 0 | 1524 | 0 | 0 | 0 | 215 | 964 | 0 | 0 | 0 | 0 | 0 | 0 | 0 | 0 | 0 |
| Carcharhinus | 0 | 0 | 0 | 0 | 751 | 4878 | 0 | 0 | 0 | 0 | 178142 | 0 | 0 | 0 | 0 | 0 | 0 | 0 |
| Carcharhinus melanopterus | 4164 | 0 | 0 | 0 | 0 | 0 | 0 | 0 | 0 | 0 | 0 | 0 | 0 | 0 | 290 | 0 | 0 | 0 |
| Centropyge | 29050 | 120114 | 141376 | 42803 | 130555 | 90445 | 25815 | 0 | 20505 | 31 | 0 | 0 | 0 | 29747 | 61990 | 44844 | 0 | 0 |
| Cephalopholis | 384 | 675 | 0 | 7579 | 0 | 29 | 0 | 0 | 391 | 0 | 0 | 0 | 0 | 1819 | 0 | 78 | 0 | 0 |
| Cephalopholis argus | 13360 | 1041 | 5988 | 16797 | 0 | 4563 | 0 | 0 | 0 | 0 | 0 | 0 | 0 | 0 | 0 | 0 | 0 | 0 |
| Cephalopholis urodeta | 9876 | 0 | 0 | 0 | 29 | 7932 | 0 | 5112 | 1872 | 0 | 0 | 0 | 0 | 0 | 0 | 5642 | 17659 | 0 |
| Cetoscarus bicolor | 95 | 0 | 0 | 1052 | 0 | 0 | 0 | 0 | 0 | 0 | 0 | 0 | 0 | 0 | 0 | 0 | 0 | 0 |
| Chaetodon | 4520 | 14 | 5630 | 71 | 13 | 0 | 0 | 0 | 114 | 0 | 0 | 0 | 0 | 0 | 0 | 14683 | 0 | 0 |
| Chaetodon kleinii | 1346 | 0 | 0 | 0 | 0 | 233 | 0 | 0 | 0 | 0 | 0 | 0 | 0 | 0 | 0 | 0 | 0 | 0 |
| Chanos chanos | 5313 | 0 | 0 | 5316 | 60111 | 0 | 4723 | 0 | 28767 | 0 | 0 | 0 | 0 | 0 | 0 | 0 | 0 | 0 |
| Cheilinus oxycephalus | 0 | 0 | 0 | 0 | 182404 | 6788 | 6902 | 0 | 0 | 0 | 0 | 0 | 0 | 0 | 0 | 0 | 0 | 0 |
| Cheilinus trilobatus | 9384 | 64983 | 337404 | 91896 | 783 | 43 | 47 | 2762 | 0 | 2232 | 0 | 0 | 0 | 0 | 111498 | 0 | 0 | 0 |
| Chromis | 347721 | 688014 | 151167 | 1980872 | 1638960 | 194546 | 327712 | 279536 | 2602946 | 1918 | 0 | 9620 | 2635 | 1151704 | 80770 | 56675 | 0 | 0 |
| Chromis viridis | 92 | 0 | 0 | 0 | 3652 | 124 | 6727 | 17911 | 8096 | 0 | 0 | 0 | 0 | 0 | 0 | 0 | 0 | 0 |
| Chrysiptera | 2124 | 0 | 51 | 434 | 0 | 425 | 4286 | 11791 | 1792 | 0 | 0 | 0 | 0 | 302 | 15 | 0 | 0 | 0 |
| Cichliformes | 0 | 18993 | 0 | 0 | 0 | 12329 | 0 | 0 | 3852 | 0 | 0 | 0 | 0 | 0 | 644633 | 0 | 0 | 0 |
| Cirrhitichthys oxycephalus | 44960 | 20616 | 64356 | 1044 | 0 | 7358 | 0 | 0 | 3364 | 0 | 0 | 0 | 0 | 22552 | 1508 | 0 | 0 | 0 |
| Cirrhitidae | 0 | 0 | 0 | 0 | 0 | 26669 | 0 | 0 | 0 | 0 | 0 | 0 | 0 | 0 | 0 | 1440 | 0 | 0 |
| Clupeiformes | 0 | 0 | 0 | 0 | 80731 | 27402 | 0 | 764 | 3048 | 0 | 0 | 0 | 0 | 0 | 0 | 0 | 0 | 0 |
| Clupeocephala | 52022 | 42320 | 258 | 636 | 935218 | 11853 | 1491 | 8917 | 25270 | 0 | 0 | 0 | 63096 | 120616 | 3505 | 48 | 0 | 0 |
| Coris | 41 | 0 | 190 | 0 | 4365 | 283 | 397 | 4065 | 53 | 280 | 0 | 0 | 0 | 0 | 4078 | 0 | 0 | 0 |
| Coryphaena equiselis | 0 | 0 | 0 | 0 | 0 | 0 | 0 | 0 | 0 | 0 | 0 | 0 | 190 | 0 | 0 | 0 | 0 | 0 |
| Crenimugil crenilabis | 82059 | 203110 | 77440 | 0 | 0 | 167 | 0 | 0 | 0 | 0 | 0 | 0 | 0 | 0 | 0 | 0 | 0 | 0 |
| Crossorhombus valderostratus | 4291 | 15157 | 0 | 0 | 0 | 0 | 0 | 0 | 0 | 0 | 0 | 0 | 0 | 0 | 0 | 0 | 0 | 0 |
| Ctenosquamata | 0 | 0 | 0 | 0 | 0 | 2652 | 0 | 0 | 0 | 0 | 0 | 0 | 0 | 0 | 0 | 0 | 0 | 0 |
| Cubiceps squamiceps | 170 | 718 | 0 | 14834 | 0 | 0 | 0 | 0 | 0 | 1480020 | 3234 | 0 | 18132 | 0 | 1273 | 0 | 0 | 0 |
| Cypriniformes | 265 | 90 | 317 | 0 | 379 | 0 | 12929 | 0 | 0 | 0 | 0 | 0 | 0 | 0 | 0 | 0 | 0 | 0 |
| Cyprinodontiformes | 0 | 0 | 0 | 0 | 0 | 565 | 269 | 0 | 16 | 0 | 0 | 0 | 0 | 0 | 0 | 0 | 0 | 0 |
| Dactyloptena orientalis | 0 | 0 | 0 | 0 | 0 | 0 | 0 | 0 | 0 | 0 | 0 | 0 | 9444 | 0 | 0 | 0 | 0 | 0 |
| Dascyllus | 0 | 0 | 0 | 12924 | 35702 | 8610 | 51428 | 3709 | 114076 | 0 | 0 | 0 | 0 | 0 | 0 | 0 | 0 | 0 |
| Dascyllus trimaculatus | 933 | 3009 | 0 | 0 | 43200 | 554 | 0 | 691 | 5855 | 0 | 0 | 0 | 0 | 0 | 0 | 0 | 0 | 0 |
| Dasyatidae | 0 | 0 | 0 | 0 | 0 | 7805 | 0 | 0 | 0 | 0 | 0 | 0 | 0 | 0 | 0 | 0 | 0 | 0 |
| Decapterus macarellus | 0 | 0 | 339 | 5734 | 2205 | 0 | 0 | 0 | 0 | 0 | 0 | 0 | 0 | 12062 | 0 | 269 | 0 | 0 |
| Diaphus | 126 | 7914 | 10084 | 774 | 320 | 6572 | 0 | 17103 | 4275 | 96700 | 215 | 14663 | 7472 | 19288 | 0 | 20 | 0 | 495 |
| Diaphus splendidus | 0 | 0 | 0 | 0 | 0 | 0 | 0 | 0 | 0 | 0 | 72205 | 0 | 255 | 0 | 0 | 346 | 0 | 0 |
| Diodon hystrix | 369 | 0 | 0 | 0 | 0 | 0 | 0 | 0 | 18 | 0 | 0 | 0 | 263 | 0 | 0 | 0 | 0 | 0 |
| Diplophos | 0 | 0 | 2235 | 0 | 0 | 0 | 0 | 0 | 0 | 0 | 0 | 0 | 0 | 278 | 0 | 1382 | 0 | 9072 |
| Echidna | 0 | 314 | 0 | 0 | 0 | 0 | 0 | 0 | 0 | 0 | 0 | 0 | 0 | 0 | 0 | 7284 | 0 | 0 |
| Elagatis bipinnulata | 0 | 0 | 0 | 0 | 0 | 6386 | 0 | 0 | 0 | 0 | 0 | 1479 | 20542 | 0 | 0 | 0 | 0 | 0 |
| Elasmobranchii | 0 | 0 | 0 | 0 | 0 | 0 | 28 | 0 | 23 | 0 | 0 | 0 | 0 | 0 | 0 | 0 | 0 | 0 |
| Encrasicholina punctifer | 0 | 3450 | 15488 | 462994 | 50949 | 0 | 1091 | 0 | 0 | 0 | 0 | 66300 | 146 | 7451 | 0 | 0 | 0 | 0 |
| Epinephelus | 85 | 228 | 76434 | 0 | 0 | 0 | 28807 | 0 | 0 | 0 | 0 | 0 | 0 | 0 | 0 | 0 | 0 | 0 |
| Epinephelus fuscoguttatus | 0 | 0 | 0 | 180 | 0 | 0 | 0 | 0 | 0 | 0 | 0 | 0 | 0 | 0 | 0 | 0 | 0 | 0 |
| Euacanthomorphacea | 573806 | 93500 | 98153 | 46743 | 176830 | 326328 | 366596 | 74614 | 138866 | 0 | 5786 | 0 | 0 | 21760 | 94470 | 437 | 0 | 0 |
| Eupercaria | 15462 | 3875 | 5778 | 0 | 2496 | 3999 | 10637 | 12730 | 8548 | 0 | 0 | 0 | 0 | 0 | 604 | 0 | 0 | 0 |
| Eurypterygia | 0 | 0 | 0 | 0 | 0 | 0 | 19750 | 0 | 974 | 0 | 0 | 0 | 0 | 0 | 0 | 1244 | 0 | 0 |
| Euteleosteomorpha | 0 | 0 | 0 | 0 | 0 | 0 | 0 | 0 | 0 | 0 | 0 | 0 | 0 | 0 | 0 | 0 | 799 | 0 |
| Exocoetidae | 148 | 0 | 2790 | 431923 | 11 | 16669 | 0 | 5138 | 7164 | 0 | 513683 | 7632602 | 13979258 | 1756 | 0 | 83076 | 241643 | 19 |
| Fistularia commersonii | 182 | 0 | 0 | 0 | 9608 | 940 | 13946 | 0 | 0 | 0 | 8694 | 0 | 0 | 0 | 0 | 0 | 0 | 0 |
| Fowleria vaiulae | 0 | 0 | 0 | 0 | 8480 | 15594 | 0 | 0 | 0 | 0 | 0 | 0 | 0 | 0 | 0 | 0 | 0 | 0 |
| Gempylus serpens | 0 | 0 | 0 | 0 | 0 | 0 | 0 | 549 | 0 | 1348 | 0 | 0 | 90852 | 0 | 0 | 25625 | 43344 | 12 |
| Gnatholepis anjerensis | 351580 | 6913 | 265 | 52761 | 0 | 81612 | 7757 | 8361 | 0 | 0 | 0 | 0 | 0 | 0 | 0 | 0 | 0 | 0 |
| Gobiidae | 1116 | 0 | 0 | 0 | 6962 | 0 | 0 | 0 | 0 | 0 | 0 | 0 | 0 | 0 | 0 | 0 | 0 | 0 |
| Gomphosus | 79629 | 22502 | 94104 | 56436 | 760362 | 40762 | 0 | 16919 | 52213 | 5539 | 8664 | 0 | 0 | 89667 | 619440 | 10665 | 0 | 0 |
| Grammistes sexlineatus | 8318 | 0 | 0 | 0 | 0 | 0 | 0 | 0 | 5828 | 0 | 0 | 0 | 0 | 0 | 0 | 0 | 0 | 0 |
| Gunnellichthys monostigma | 0 | 0 | 0 | 0 | 2016 | 38132 | 13548 | 2513 | 1960 | 0 | 0 | 0 | 0 | 0 | 0 | 0 | 0 | 0 |
| Gymnothorax | 831 | 0 | 7378 | 0 | 0 | 2687 | 0 | 0 | 679 | 0 | 0 | 0 | 0 | 0 | 733 | 100132 | 0 | 0 |
| Gymnothorax flavimarginatus | 18392 | 0 | 10270 | 10979 | 17611 | 0 | 51013 | 14340 | 19326 | 0 | 0 | 0 | 0 | 0 | 8570 | 0 | 0 | 0 |
| Halichoeres | 1053 | 0 | 0 | 4176 | 47143 | 517 | 70007 | 5748 | 4658 | 0 | 0 | 0 | 0 | 0 | 896 | 0 | 0 | 0 |
| Halichoeres hortulanus | 5564 | 0 | 24734 | 0 | 7011 | 975 | 4457 | 0 | 0 | 0 | 34535 | 0 | 0 | 0 | 7003 | 0 | 0 | 0 |
| Halichoeres scapularis | 323961 | 12048 | 158070 | 3680 | 22518 | 68798 | 61856 | 173408 | 45471 | 0 | 0 | 0 | 0 | 4112 | 1204485 | 0 | 0 | 0 |
| Hemigymnus | 0 | 0 | 0 | 0 | 0 | 0 | 0 | 0 | 494 | 0 | 0 | 36053 | 3497 | 0 | 0 | 0 | 0 | 0 |
| Hemiramphidae | 0 | 0 | 31 | 0 | 0 | 0 | 0 | 0 | 0 | 0 | 0 | 0 | 0 | 0 | 0 | 0 | 0 | 0 |
| Hemiramphus | 0 | 0 | 38471 | 20503 | 0 | 0 | 0 | 0 | 13213 | 210 | 0 | 13309 | 685325 | 0 | 0 | 0 | 4744 | 0 |
| Heteroconger hassi | 0 | 0 | 0 | 0 | 1784 | 3124 | 0 | 0 | 0 | 0 | 0 | 0 | 2818 | 0 | 0 | 0 | 0 | 0 |
| Himantura | 0 | 0 | 0 | 0 | 28630 | 297 | 0 | 0 | 265740 | 0 | 0 | 0 | 0 | 0 | 0 | 0 | 0 | 0 |
| Holocentridae | 5400 | 0 | 587 | 5950 | 160467 | 1740 | 0 | 0 | 0 | 0 | 0 | 0 | 3130 | 0 | 0 | 1120 | 0 | 0 |
| Holocentriformes | 410 | 0 | 0 | 0 | 0 | 0 | 0 | 0 | 0 | 0 | 0 | 0 | 0 | 0 | 0 | 0 | 0 | 0 |
| Iniistius | 0 | 0 | 0 | 0 | 21876 | 52 | 0 | 1452 | 2942 | 0 | 0 | 0 | 0 | 0 | 0 | 0 | 0 | 0 |
| Katsuwonus pelamis | 2706 | 0 | 3424 | 16780 | 0 | 180 | 3667 | 0 | 2485 | 33870 | 349536 | 0 | 1528252 | 477 | 0 | 6156 | 115020 | 0 |
| Kyphosus | 21301 | 8835 | 12450 | 5142 | 0 | 0 | 0 | 0 | 0 | 0 | 0 | 0 | 0 | 22182 | 2506 | 0 | 0 | 0 |
| Kyphosus cinerascens | 7656 | 8645 | 5410 | 2646 | 116145 | 100636 | 25634 | 0 | 74331 | 0 | 0 | 0 | 0 | 67669 | 8212 | 0 | 0 | 0 |
| Kyphosus vaigiensis | 21593 | 0 | 47916 | 68223 | 58447 | 11807 | 0 | 0 | 0 | 0 | 0 | 0 | 0 | 0 | 0 | 0 | 0 | 0 |
| Labridae | 489588 | 829917 | 1744737 | 1429087 | 30403 | 20557 | 17730 | 28551 | 133320 | 0 | 127892 | 0 | 0 | 0 | 380044 | 124891 | 6799 | 40 |
| Labriformes | 0 | 0 | 0 | 0 | 257664 | 15672 | 70489 | 0 | 17029 | 0 | 0 | 0 | 0 | 0 | 0 | 0 | 0 | 0 |
| Labroides | 0 | 710 | 17225 | 0 | 0 | 3451 | 0 | 0 | 9714 | 0 | 0 | 0 | 0 | 0 | 6807 | 0 | 0 | 0 |
| Lagocephalus lagocephalus | 0 | 0 | 0 | 0 | 0 | 0 | 11 | 0 | 0 | 0 | 0 | 0 | 0 | 0 | 0 | 0 | 0 | 0 |
| Lethrinus | 12324 | 556 | 0 | 0 | 10348 | 4576 | 0 | 886 | 55882 | 0 | 0 | 0 | 0 | 0 | 1573 | 0 | 0 | 0 |
| Lutjanus | 43799 | 0 | 30801 | 29563 | 460363 | 297356 | 26408 | 43919 | 81209 | 0 | 37509 | 0 | 3584 | 6503 | 4239 | 449781 | 266411 | 33 |
| Macropharyngodon bipartitus | 0 | 13254 | 1967 | 0 | 8875 | 74 | 0 | 6767 | 0 | 0 | 0 | 0 | 0 | 0 | 0 | 84 | 0 | 0 |
| Melichthys | 0 | 0 | 0 | 0 | 0 | 0 | 0 | 0 | 0 | 0 | 17803 | 0 | 0 | 0 | 0 | 4050 | 0 | 0 |
| Mobula tarapacana | 0 | 0 | 0 | 0 | 1206 | 0 | 0 | 0 | 0 | 0 | 0 | 0 | 0 | 0 | 0 | 0 | 0 | 0 |
| Monodactylus | 0 | 1682 | 0 | 11630 | 0 | 0 | 0 | 0 | 0 | 0 | 0 | 0 | 0 | 0 | 0 | 0 | 0 | 0 |
| Monotaxis | 0 | 0 | 0 | 0 | 0 | 0 | 0 | 0 | 16 | 0 | 161315 | 0 | 0 | 0 | 0 | 41 | 0 | 0 |
| Moringua | 0 | 0 | 0 | 0 | 0 | 296 | 0 | 0 | 0 | 0 | 0 | 0 | 0 | 0 | 0 | 40 | 0 | 0 |
| Mugilidae | 0 | 0 | 0 | 0 | 0 | 15 | 0 | 0 | 0 | 0 | 0 | 0 | 0 | 0 | 0 | 0 | 0 | 0 |
| Mugiliformes | 0 | 33 | 0 | 0 | 0 | 0 | 0 | 0 | 3701 | 0 | 0 | 0 | 0 | 0 | 0 | 0 | 34987 | 72576 |
| Mullidae | 0 | 10597 | 1521 | 0 | 4478 | 1662 | 0 | 39256 | 0 | 0 | 0 | 0 | 0 | 0 | 0 | 0 | 0 | 0 |
| Mulloidichthys | 7500 | 654 | 3193 | 0 | 4511 | 1672 | 0 | 0 | 0 | 0 | 0 | 0 | 12411 | 0 | 0 | 11398 | 0 | 0 |
| Muraenidae | 1086 | 115 | 0 | 18031 | 15881 | 16682 | 6649 | 0 | 25174 | 0 | 0 | 0 | 0 | 0 | 0 | 77 | 0 | 0 |
| Myctophidae | 4963 | 9273 | 5456 | 22842 | 6515 | 664 | 0 | 11129 | 8270 | 419676 | 49020 | 0 | 34365 | 71979 | 0 | 75279 | 100676 | 48454 |
| Myctophiformes | 18 | 0 | 0 | 0 | 0 | 0 | 8954 | 0 | 0 | 0 | 0 | 0 | 662 | 67839 | 0 | 532 | 2579 | 12 |
| Myctophum | 0 | 0 | 0 | 0 | 2824 | 18028 | 12 | 5085 | 0 | 0 | 0 | 0 | 6956 | 0 | 0 | 4126 | 0 | 0 |
| Myliobatiformes | 0 | 0 | 0 | 0 | 0 | 0 | 0 | 0 | 246 | 0 | 0 | 0 | 0 | 0 | 0 | 0 | 0 | 0 |
| Myripristis | 25863 | 0 | 0 | 6508 | 65347 | 110670 | 0 | 50938 | 28704 | 0 | 0 | 0 | 65 | 0 | 10510 | 0 | 0 | 0 |
| Myripristis berndti | 468 | 0 | 4699 | 14038 | 10300 | 91 | 0 | 0 | 5186 | 0 | 0 | 0 | 15013 | 870 | 12816 | 0 | 0 | 0 |
| Naso | 146787 | 1073856 | 1021984 | 10358 | 137809 | 226540 | 172949 | 443706 | 111978 | 427815 | 0 | 70497 | 0 | 438659 | 81783 | 357871 | 0 | 3634 |
| Nealotus tripes | 848 | 0 | 0 | 0 | 0 | 0 | 0 | 0 | 0 | 0 | 0 | 0 | 0 | 0 | 0 | 0 | 0 | 0 |
| Nemateleotris | 0 | 0 | 0 | 16000 | 0 | 0 | 0 | 0 | 0 | 0 | 0 | 0 | 0 | 0 | 0 | 0 | 0 | 0 |
| Notoscopelus caudispinosus | 0 | 0 | 0 | 1012 | 0 | 0 | 0 | 0 | 0 | 0 | 0 | 0 | 0 | 0 | 0 | 0 | 0 | 0 |
| Novaculichthys | 2970 | 0 | 113 | 0 | 49 | 2400 | 2009 | 58761 | 729 | 0 | 0 | 0 | 0 | 0 | 58 | 14 | 0 | 0 |
| Odonus niger | 0 | 0 | 0 | 0 | 4335 | 6105 | 6657 | 10734 | 49370 | 113 | 0 | 0 | 0 | 0 | 0 | 0 | 0 | 0 |
| Ophiocara | 1258 | 3678 | 48364 | 0 | 0 | 0 | 0 | 0 | 0 | 0 | 0 | 0 | 0 | 0 | 0 | 0 | 0 | 0 |
| Ostichthys | 0 | 0 | 0 | 0 | 0 | 0 | 1993 | 0 | 0 | 0 | 0 | 0 | 0 | 0 | 0 | 0 | 0 | 37 |
| Ostorhinchus | 59 | 0 | 0 | 0 | 0 | 4056 | 0 | 0 | 37643 | 0 | 0 | 0 | 0 | 0 | 0 | 0 | 0 | 0 |
| Ostorhinchus apogonoides | 0 | 3536 | 5635 | 0 | 140466 | 28584 | 0 | 0 | 1034 | 0 | 0 | 0 | 0 | 0 | 1818 | 0 | 0 | 0 |
| Ostorhinchus taeniophorus | 0 | 0 | 955 | 0 | 0 | 1548 | 0 | 2207 | 40987 | 0 | 0 | 0 | 0 | 0 | 4275 | 0 | 0 | 0 |
| Ostraciidae | 1370 | 0 | 0 | 0 | 0 | 0 | 0 | 0 | 0 | 0 | 0 | 0 | 0 | 0 | 1920 | 0 | 0 | 0 |
| Oxycheilinus | 66 | 0 | 0 | 0 | 4661 | 9714 | 0 | 51678 | 10068 | 0 | 0 | 0 | 0 | 0 | 0 | 0 | 0 | 0 |
| Paracirrhites arcatus | 0 | 0 | 647 | 303 | 3228 | 37130 | 0 | 19216 | 144315 | 0 | 0 | 0 | 0 | 0 | 0 | 10956 | 0 | 0 |
| Parapercis hexophtalma | 0 | 0 | 109 | 0 | 0 | 0 | 0 | 0 | 0 | 0 | 0 | 0 | 0 | 0 | 0 | 0 | 0 | 0 |
| Parexocoetus | 0 | 0 | 0 | 0 | 2581 | 305 | 0 | 34782 | 0 | 0 | 0 | 0 | 0 | 0 | 0 | 0 | 0 | 0 |
| Parupeneus | 78730 | 17037 | 28816 | 76762 | 44304 | 53728 | 76769 | 21835 | 209217 | 365977 | 0 | 0 | 205 | 0 | 0 | 11579 | 6055 | 14 |
| Pastinachus atrus | 0 | 0 | 0 | 0 | 0 | 2549 | 0 | 0 | 0 | 0 | 0 | 0 | 0 | 0 | 0 | 0 | 0 | 0 |
| Pateobatis | 1156 | 10092 | 11837 | 0 | 1456 | 91578 | 47533 | 6003 | 878048 | 0 | 0 | 0 | 0 | 0 | 0 | 0 | 0 | 0 |
| Pempheriformes | 0 | 0 | 0 | 0 | 0 | 0 | 0 | 0 | 0 | 0 | 0 | 0 | 0 | 0 | 0 | 3826 | 0 | 0 |
| Pempheris | 0 | 374 | 0 | 0 | 0 | 13675 | 40689 | 0 | 358 | 119278 | 0 | 0 | 0 | 22022 | 9992 | 0 | 0 | 0 |
| Perciformes | 774 | 23 | 123 | 0 | 0 | 8583 | 0 | 0 | 1155 | 0 | 0 | 0 | 0 | 0 | 0 | 1816 | 0 | 0 |
| Percomorphaceae | 346481 | 344384 | 171130 | 118280 | 292759 | 108104 | 297018 | 71031 | 113057 | 0 | 363613 | 0 | 0 | 97094 | 27417 | 99488 | 0 | 0 |
| Plectorhinchus | 161590 | 116324 | 176064 | 2966 | 10079 | 98948 | 28876 | 2163 | 648 | 0 | 0 | 0 | 0 | 0 | 1787 | 0 | 0 | 0 |
| Plectroglyphidodon | 16386 | 0 | 20257 | 938 | 26246 | 17189 | 0 | 40648 | 4287 | 0 | 0 | 0 | 0 | 17515 | 2134 | 1054 | 0 | 0 |
| Plectroglyphidodon leucozonus | 852 | 0 | 0 | 0 | 0 | 0 | 0 | 0 | 0 | 0 | 0 | 0 | 0 | 7160 | 0 | 679 | 0 | 0 |
| Pleuronectiformes | 1695 | 0 | 0 | 0 | 0 | 0 | 0 | 0 | 0 | 0 | 0 | 0 | 0 | 0 | 0 | 0 | 0 | 0 |
| Pomacanthus imperator | 0 | 0 | 0 | 0 | 0 | 0 | 106149 | 0 | 0 | 0 | 0 | 0 | 0 | 0 | 0 | 0 | 0 | 0 |
| Pomacentridae | 22253 | 80293 | 1929 | 18591 | 54436 | 1064 | 17779 | 18437 | 35247 | 6140 | 7355 | 0 | 0 | 314 | 6280 | 410594 | 0 | 13623 |
| Pristiapogon | 0 | 0 | 0 | 0 | 1377 | 566 | 0 | 1342 | 710 | 0 | 0 | 0 | 0 | 0 | 0 | 0 | 0 | 0 |
| Pristiapogon kallopterus | 30 | 98 | 0 | 0 | 0 | 2762 | 174 | 0 | 0 | 0 | 0 | 0 | 0 | 0 | 0 | 0 | 0 | 0 |
| Prognichthys sealei | 0 | 0 | 0 | 0 | 0 | 0 | 0 | 0 | 0 | 0 | 0 | 0 | 54949 | 0 | 0 | 0 | 0 | 0 |
| Pseudanthias | 26800 | 6918 | 71344 | 182025 | 716489 | 12789 | 38742 | 92906 | 62434 | 0 | 17523 | 0 | 0 | 297216 | 580 | 47844 | 0 | 0 |
| Pseudanthias squamipinnis | 69648 | 35463 | 10111 | 24597 | 141382 | 300570 | 267688 | 34298 | 299400 | 284 | 0 | 0 | 0 | 12007 | 61290 | 341383 | 5545 | 43 |
| Pseudobalistes flavimarginatus | 126370 | 97230 | 32270 | 10966 | 112284 | 13277 | 24255 | 0 | 18971 | 0 | 0 | 0 | 0 | 0 | 1891 | 10486 | 0 | 0 |
| Pseudocheilinus | 13390 | 0 | 15088 | 6692 | 79653 | 84714 | 7569 | 60820 | 5075 | 0 | 0 | 0 | 0 | 10535 | 7534 | 7666 | 0 | 0 |
| Pseudocheilinus evanidus | 0 | 0 | 0 | 1055 | 72654 | 36 | 10929 | 0 | 273 | 0 | 0 | 0 | 0 | 0 | 0 | 0 | 0 | 0 |
| Pseudocoris | 0 | 0 | 0 | 0 | 0 | 867 | 58 | 688 | 0 | 0 | 87 | 0 | 0 | 0 | 0 | 0 | 0 | 0 |
| Ptereleotris | 3288 | 0 | 0 | 4976 | 37350 | 35860 | 14878 | 456 | 14767 | 0 | 0 | 0 | 0 | 3143 | 4055 | 70878 | 0 | 0 |
| Ptereleotris heteroptera | 0 | 0 | 0 | 0 | 11823 | 0 | 0 | 0 | 624 | 0 | 0 | 63104 | 0 | 0 | 0 | 0 | 0 | 0 |
| Pterocaesio | 0 | 0 | 46 | 0 | 0 | 0 | 0 | 0 | 0 | 0 | 0 | 0 | 0 | 0 | 0 | 14707 | 0 | 0 |
| Pterocaesio tile | 31738 | 1096 | 19827 | 96160 | 12389 | 3298 | 290 | 7282 | 1792 | 0 | 0 | 0 | 0 | 49734 | 6756 | 156209 | 0 | 0 |
| Pygoplites diacanthus | 0 | 0 | 4253 | 0 | 7042 | 0 | 0 | 0 | 0 | 0 | 0 | 0 | 0 | 0 | 0 | 0 | 0 | 0 |
| Rhabdamia | 0 | 0 | 0 | 0 | 337 | 12216 | 0 | 19964 | 788 | 0 | 0 | 0 | 0 | 0 | 0 | 0 | 0 | 0 |
| Rhinecanthus | 74370 | 117 | 14514 | 461 | 0 | 3819 | 44037 | 0 | 3215 | 0 | 0 | 0 | 0 | 13550 | 0 | 53826 | 0 | 0 |
| Rhinecanthus aculeatus | 114256 | 67836 | 225662 | 36768 | 41700 | 12037 | 82755 | 1299 | 124626 | 0 | 0 | 0 | 0 | 2494 | 751 | 108 | 0 | 0 |
| Salarias | 2804 | 0 | 495 | 0 | 0 | 0 | 0 | 0 | 0 | 0 | 0 | 0 | 0 | 0 | 0 | 0 | 0 | 0 |
| Salmoniformes | 0 | 0 | 0 | 0 | 0 | 48 | 0 | 0 | 0 | 0 | 0 | 0 | 77 | 0 | 0 | 0 | 0 | 0 |
| Sargocentron | 1796 | 0 | 2428 | 3123 | 0 | 1258 | 0 | 0 | 26338 | 0 | 0 | 0 | 0 | 0 | 0 | 0 | 0 | 0 |
| Sargocentron diadema | 0 | 0 | 19 | 0 | 42725 | 20884 | 0 | 0 | 1501 | 0 | 0 | 0 | 52190 | 0 | 0 | 0 | 0 | 0 |
| Sargocentron spiniferum | 5995 | 4554 | 2515 | 0 | 0 | 487 | 0 | 0 | 0 | 0 | 0 | 0 | 0 | 0 | 0 | 660 | 0 | 0 |
| Scarus rubroviolaceus | 480312 | 50799 | 106464 | 98210 | 252240 | 126977 | 38548 | 194388 | 292406 | 0 | 219074 | 0 | 0 | 85308 | 234520 | 4259 | 0 | 0 |
| Scombridae | 0 | 0 | 0 | 0 | 0 | 0 | 0 | 0 | 0 | 0 | 0 | 0 | 3057 | 246 | 0 | 0 | 0 | 0 |
| Scopelosaurus | 0 | 0 | 0 | 0 | 0 | 0 | 0 | 2342 | 0 | 0 | 0 | 0 | 0 | 0 | 0 | 0 | 0 | 12 |
| Scuticaria tigrina | 0 | 0 | 0 | 0 | 0 | 5114 | 0 | 0 | 0 | 0 | 0 | 0 | 0 | 0 | 0 | 0 | 0 | 0 |
| Serranidae | 0 | 0 | 0 | 5234 | 2526 | 1325 | 0 | 0 | 0 | 0 | 0 | 0 | 0 | 0 | 3730 | 839 | 0 | 0 |
| Siganus | 0 | 0 | 0 | 0 | 0 | 978 | 0 | 0 | 0 | 0 | 0 | 0 | 0 | 0 | 0 | 0 | 0 | 0 |
| Siluriformes | 12 | 0 | 68 | 0 | 0 | 0 | 0 | 0 | 0 | 0 | 0 | 0 | 0 | 0 | 0 | 0 | 0 | 0 |
| Sphyraena | 1576 | 0 | 0 | 0 | 0 | 0 | 0 | 0 | 15 | 0 | 0 | 0 | 0 | 0 | 0 | 0 | 0 | 0 |
| Sphyraenidae | 0 | 0 | 0 | 0 | 762 | 12 | 0 | 0 | 0 | 0 | 0 | 0 | 0 | 0 | 0 | 0 | 0 | 0 |
| Sphyrna sp. MV-2009 | 0 | 0 | 0 | 34 | 0 | 0 | 0 | 0 | 0 | 0 | 0 | 0 | 0 | 0 | 0 | 0 | 0 | 0 |
| Spratelloides | 0 | 3560 | 0 | 0 | 115850 | 637227 | 60181 | 258228 | 256028 | 0 | 0 | 0 | 0 | 0 | 0 | 0 | 0 | 0 |
| Stethojulis | 306632 | 331299 | 897227 | 346832 | 30432 | 42066 | 5973 | 27214 | 86969 | 0 | 0 | 0 | 0 | 62953 | 152625 | 0 | 0 | 0 |
| Stomias | 0 | 0 | 0 | 0 | 0 | 0 | 0 | 0 | 0 | 0 | 0 | 0 | 0 | 0 | 0 | 0 | 163 | 19 |
| Stomiidae | 0 | 0 | 0 | 3100 | 0 | 0 | 0 | 0 | 0 | 0 | 0 | 0 | 0 | 0 | 0 | 0 | 0 | 0 |
| Sufflamen chrysopterum | 0 | 403 | 0 | 0 | 43544 | 302 | 3170 | 0 | 906 | 0 | 0 | 0 | 0 | 0 | 0 | 4400 | 0 | 0 |
| Syngnathiformes | 0 | 0 | 0 | 0 | 21151 | 0 | 11670 | 22034 | 3244 | 0 | 0 | 0 | 0 | 0 | 0 | 0 | 0 | 0 |
| Synodontidae | 0 | 0 | 0 | 0 | 169 | 72 | 0 | 0 | 4973 | 0 | 0 | 0 | 0 | 0 | 0 | 0 | 0 | 0 |
| Synodus variegatus | 96 | 0 | 0 | 0 | 37641 | 43 | 710 | 0 | 6610 | 0 | 0 | 0 | 0 | 3601 | 0 | 0 | 0 | 0 |
| Taeniura | 0 | 0 | 0 | 0 | 9933 | 2641 | 0 | 2901 | 2694 | 0 | 0 | 0 | 0 | 0 | 0 | 0 | 0 | 0 |
| Teleostei | 0 | 0 | 0 | 14418 | 0 | 0 | 0 | 0 | 0 | 0 | 0 | 0 | 0 | 0 | 0 | 0 | 0 | 0 |
| Tetraodontiformes | 0 | 0 | 0 | 0 | 0 | 0 | 0 | 996 | 55 | 0 | 0 | 0 | 0 | 0 | 0 | 0 | 0 | 0 |
| Thalassoma | 417705 | 565466 | 1032262 | 385602 | 329014 | 338841 | 28634 | 46175 | 134181 | 260 | 0 | 0 | 0 | 706542 | 961577 | 97393 | 0 | 0 |
| Thalassoma amblycephalum | 697086 | 851829 | 648216 | 819196 | 19750 | 10528 | 87180 | 0 | 8860 | 0 | 0 | 0 | 0 | 6178068 | 1937773 | 115724 | 40198 | 2973522 |
| Thalassoma hardwicke | 242472 | 65428 | 233177 | 23688 | 6177 | 23305 | 0 | 0 | 1184 | 0 | 0 | 0 | 0 | 61008 | 5267883 | 0 | 0 | 0 |
| Thunnus | 0 | 0 | 0 | 0 | 0 | 1112 | 0 | 0 | 0 | 0 | 170502 | 80082 | 8317980 | 0 | 0 | 11880 | 9088 | 12 |
| Triphoturus | 0 | 22601 | 0 | 0 | 0 | 0 | 0 | 0 | 0 | 3424 | 0 | 0 | 24861 | 18214 | 0 | 271 | 0 | 13 |
| Tylosurus | 5604 | 1156 | 23370 | 182869 | 241110 | 26205 | 44 | 0 | 93702 | 0 | 38 | 24 | 287596 | 14809 | 215 | 11930 | 0 | 0 |
| Tylosurus acus | 591 | 92601 | 18980 | 250704 | 498412 | 60641 | 22040 | 83683 | 16402 | 0 | 442406 | 2629528 | 254244 | 23244 | 6564 | 380686 | 1850470 | 353112 |
| Uranoscopidae | 0 | 0 | 0 | 0 | 0 | 0 | 0 | 0 | 0 | 0 | 0 | 0 | 10377 | 0 | 0 | 0 | 0 | 0 |
| Urogymnus asperrimus | 0 | 0 | 0 | 0 | 81506 | 317541 | 60414 | 20052 | 827372 | 0 | 0 | 0 | 0 | 0 | 0 | 0 | 0 | 0 |
| Valenciennea | 0 | 0 | 0 | 0 | 1182 | 1044 | 0 | 0 | 0 | 0 | 0 | 0 | 0 | 0 | 159 | 0 | 0 | 0 |
| Variola | 0 | 0 | 0 | 0 | 5698 | 0 | 0 | 1486 | 0 | 0 | 0 | 0 | 0 | 0 | 0 | 0 | 0 | 0 |
| Wetmorella | 0 | 0 | 0 | 0 | 14 | 0 | 0 | 0 | 1011 | 0 | 0 | 0 | 0 | 0 | 0 | 0 | 0 | 0 |
| Zanclus cornutus | 1504 | 0 | 0 | 0 | 3451 | 31987 | 109754 | 61 | 57013 | 0 | 72432 | 0 | 4992 | 0 | 0 | 737 | 0 | 0 |
| Zebrasoma | 0 | 0 | 0 | 0 | 76587 | 0 | 0 | 0 | 0 | 0 | 0 | 0 | 0 | 0 | 5692 | 0 | 0 | 0 |
| Zebrasoma desjardinii | 0 | 0 | 0 | 0 | 0 | 2868 | 0 | 0 | 0 | 0 | 0 | 0 | 0 | 0 | 0 | 0 | 0 | 0 |

**Table S2:** Number of sequences detected per taxa using the “Motu pipeline” at the site levels.

|  | europa_0m | europa_300m | europa _50m | europa _600m | glorieuse | glorieuse _0m | glorieuse _100m | glorieuse _300m | glorieuse_50m | juan_0 | juan_1 | juan_2 | juan_3 | taaf_station | taaf_station2 | tromelin_0m | tromelin_100m | tromelin _50m |
| --- | --- | --- | --- | --- | --- | --- | --- | --- | --- | --- | --- | --- | --- | --- | --- | --- | --- | --- |
| Abudefduf | 299820 | 0 | 15858 | 15807 | 50917 | 252703 | 18670 | 0 | 131026 | 0 | 0 | 0 | 0 | 135844 | 850 | 592 | 0 | 0 |
| Abudefduf notatus | 216 | 0 | 2330 | 673 | 0 | 0 | 0 | 0 | 0 | 0 | 0 | 0 | 0 | 0 | 0 | 0 | 0 | 0 |
| Abudefduf septemfasciatus | 1916 | 4254 | 757 | 0 | 0 | 0 | 0 | 0 | 18518 | 0 | 0 | 0 | 0 | 0 | 5559 | 0 | 0 | 0 |
| Acanthuridae | 170667 | 42716 | 88243 | 118493 | 617645 | 321422 | 96488 | 30919 | 237654 | 48562 | 198954 | 0 | 0 | 430938 | 75087 | 10713 | 0 | 15225 |
| Acanthurus | 942718 | 794944 | 2406437 | 223695 | 208629 | 328242 | 225732 | 11699 | 343809 | 133 | 0 | 5895 | 0 | 408338 | 738770 | 101349 | 0 | 0 |
| Acanthurus bariene | 0 | 0 | 0 | 0 | 0 | 0 | 0 | 0 | 843 | 0 | 0 | 0 | 0 | 0 | 0 | 0 | 0 | 0 |
| Acanthurus lineatus | 114851 | 13660 | 90588 | 32124 | 317614 | 178376 | 23914 | 4783 | 25405 | 2033 | 554121 | 0 | 0 | 0 | 44132 | 0 | 0 | 0 |
| Acanthurus nigrofuscus | 272625 | 209356 | 294054 | 1457 | 416944 | 83988 | 277708 | 0 | 202284 | 42 | 25136 | 0 | 1108 | 17 | 456 | 95232 | 0 | 35 |
| Aethaloperca rogaa | 299 | 0 | 0 | 0 | 0 | 0 | 15388 | 0 | 2943 | 0 | 99162 | 0 | 2386 | 0 | 3386 | 198985 | 47786 | 0 |
| Albula | 185 | 0 | 0 | 0 | 0 | 21754 | 3572 | 0 | 27802 | 0 | 0 | 0 | 0 | 0 | 0 | 0 | 0 | 0 |
| Alepocephaliformes | 0 | 0 | 0 | 0 | 7599 | 0 | 0 | 0 | 0 | 0 | 0 | 0 | 0 | 0 | 0 | 73073 | 8873 | 0 |
| Amphiprion akallopisos | 0 | 0 | 0 | 0 | 12 | 167 | 0 | 0 | 0 | 0 | 0 | 0 | 0 | 0 | 0 | 0 | 0 | 0 |
| Anampses | 0 | 0 | 0 | 0 | 71 | 3066 | 0 | 0 | 2580 | 0 | 0 | 0 | 0 | 0 | 0 | 0 | 0 | 0 |
| Anguilliformes | 1284 | 17617 | 0 | 0 | 7157 | 1557 | 32 | 1973 | 0 | 0 | 0 | 0 | 0 | 0 | 5432 | 0 | 0 | 0 |
| Aphaniidae | 0 | 0 | 0 | 0 | 0 | 0 | 0 | 0 | 0 | 0 | 0 | 0 | 0 | 0 | 0 | 64840 | 0 | 0 |
| Apogon semiornatus | 0 | 0 | 0 | 0 | 21128 | 35 | 0 | 0 | 419 | 0 | 0 | 0 | 0 | 0 | 0 | 0 | 0 | 0 |
| Apolemichthys armitagei | 0 | 0 | 0 | 0 | 0 | 0 | 0 | 1730 | 0 | 0 | 0 | 0 | 0 | 0 | 0 | 0 | 0 | 0 |
| Ariosoma | 4920 | 0 | 0 | 0 | 0 | 0 | 0 | 0 | 0 | 0 | 0 | 0 | 0 | 0 | 0 | 0 | 0 | 0 |
| Arothron | 321 | 357562 | 0 | 0 | 10214 | 13572 | 83788 | 90722 | 13160 | 0 | 0 | 0 | 0 | 12523 | 0 | 0 | 0 | 0 |
| Arothron mappa | 0 | 0 | 0 | 0 | 6575 | 0 | 0 | 0 | 1537 | 0 | 0 | 0 | 0 | 0 | 0 | 0 | 0 | 0 |
| Asterropteryx | 0 | 0 | 0 | 0 | 0 | 0 | 12552 | 0 | 0 | 0 | 0 | 0 | 0 | 0 | 0 | 0 | 0 | 0 |
| Atherinidae | 45624 | 83424 | 7190 | 0 | 7750 | 42102 | 3940 | 0 | 1260 | 0 | 0 | 0 | 0 | 0 | 0 | 0 | 0 | 0 |
| Atherinomorphae | 0 | 0 | 0 | 0 | 636408 | 57127 | 22896 | 1532 | 47382 | 0 | 0 | 0 | 0 | 29 | 0 | 174 | 0 | 0 |
| Auxis | 0 | 0 | 0 | 0 | 0 | 0 | 0 | 0 | 0 | 570 | 0 | 0 | 15862 | 0 | 0 | 0 | 0 | 0 |
| Balistapus undulatus | 1095 | 648 | 86791 | 6454 | 84869 | 4502 | 14751 | 0 | 12924 | 0 | 40398 | 0 | 0 | 0 | 0 | 0 | 0 | 0 |
| Balistoides conspicillum | 0 | 0 | 0 | 0 | 0 | 0 | 0 | 0 | 688 | 0 | 0 | 0 | 0 | 0 | 0 | 555 | 0 | 0 |
| Belonidae | 188556 | 13456 | 6810 | 2566 | 3321 | 1352 | 0 | 0 | 0 | 0 | 0 | 0 | 0 | 0 | 92125 | 113926 | 0 | 0 |
| Benthosema | 250 | 0 | 0 | 0 | 952 | 107 | 24949 | 9374 | 11368 | 0 | 0 | 0 | 0 | 0 | 0 | 0 | 0 | 0 |
| Blenniiformes | 0 | 0 | 0 | 4730 | 0 | 0 | 0 | 0 | 0 | 255731 | 0 | 0 | 0 | 0 | 0 | 0 | 0 | 0 |
| Bolinichthys | 2964 | 0 | 0 | 0 | 73466 | 0 | 0 | 4251 | 0 | 37703 | 1129 | 36196 | 9312 | 0 | 408 | 46177 | 0 | 915 |
| Caesio | 1286 | 12401 | 3676 | 348 | 96289 | 36403 | 13947 | 24465 | 23560 | 0 | 6636 | 0 | 0 | 27672 | 23717 | 13689 | 0 | 0 |
| Caesio teres | 11284 | 40548 | 862 | 37728 | 281349 | 45018 | 133862 | 50151 | 5523 | 639648 | 72112 | 0 | 0 | 717 | 14688 | 0 | 0 | 0 |
| Cantherhines pardalis | 0 | 11649 | 0 | 0 | 0 | 0 | 0 | 0 | 2752 | 0 | 100885 | 24354 | 1270 | 0 | 0 | 4788 | 0 | 0 |
| Canthidermis maculata | 0 | 0 | 0 | 0 | 0 | 0 | 0 | 0 | 5297 | 13 | 0 | 0 | 0 | 0 | 0 | 0 | 0 | 0 |
| Canthigaster | 0 | 0 | 576 | 1978 | 0 | 15277 | 0 | 18423 | 6962 | 0 | 0 | 0 | 0 | 0 | 0 | 0 | 0 | 0 |
| Canthigaster valentini | 0 | 0 | 2005 | 0 | 0 | 0 | 0 | 0 | 0 | 0 | 0 | 0 | 0 | 0 | 0 | 0 | 0 | 0 |
| Carangidae | 0 | 0 | 0 | 0 | 0 | 24581 | 0 | 0 | 19 | 0 | 0 | 0 | 0 | 0 | 0 | 0 | 0 | 0 |
| Caranx | 0 | 0 | 0 | 0 | 0 | 0 | 0 | 0 | 0 | 0 | 0 | 0 | 0 | 0 | 0 | 0 | 3581 | 0 |
| Caranx melampygus | 342 | 0 | 0 | 0 | 1675 | 1511 | 52585 | 347 | 5286 | 0 | 0 | 0 | 0 | 52 | 0 | 4164 | 12 | 3107 |
| Carcharhinidae | 30 | 0 | 0 | 1524 | 0 | 0 | 0 | 215 | 964 | 0 | 0 | 0 | 0 | 0 | 0 | 0 | 0 | 0 |
| Carcharhinus | 0 | 0 | 0 | 0 | 751 | 4878 | 0 | 0 | 0 | 0 | 178142 | 0 | 0 | 0 | 0 | 0 | 0 | 0 |
| Carcharhinus melanopterus | 4164 | 0 | 0 | 0 | 0 | 0 | 0 | 0 | 0 | 0 | 0 | 0 | 0 | 0 | 290 | 0 | 0 | 0 |
| Centropyge | 29050 | 120114 | 141376 | 42803 | 130555 | 90445 | 25815 | 0 | 20505 | 31 | 0 | 0 | 0 | 29747 | 61990 | 44844 | 0 | 0 |
| Cephalopholis | 384 | 675 | 0 | 7579 | 0 | 29 | 0 | 0 | 391 | 0 | 0 | 0 | 0 | 1819 | 0 | 78 | 0 | 0 |
| Cephalopholis argus | 13360 | 1041 | 5988 | 16797 | 0 | 4563 | 0 | 0 | 0 | 0 | 0 | 0 | 0 | 0 | 0 | 0 | 0 | 0 |
| Cephalopholis urodeta | 9876 | 0 | 0 | 0 | 29 | 7932 | 0 | 5112 | 1872 | 0 | 0 | 0 | 0 | 0 | 0 | 5642 | 17659 | 0 |
| Cetoscarus bicolor | 95 | 0 | 0 | 1052 | 0 | 0 | 0 | 0 | 0 | 0 | 0 | 0 | 0 | 0 | 0 | 0 | 0 | 0 |
| Chaetodon | 4520 | 14 | 5630 | 71 | 13 | 0 | 0 | 0 | 114 | 0 | 0 | 0 | 0 | 0 | 0 | 14683 | 0 | 0 |
| Chaetodon kleinii | 1346 | 0 | 0 | 0 | 0 | 233 | 0 | 0 | 0 | 0 | 0 | 0 | 0 | 0 | 0 | 0 | 0 | 0 |
| Chanos chanos | 5313 | 0 | 0 | 5316 | 60111 | 0 | 4723 | 0 | 28767 | 0 | 0 | 0 | 0 | 0 | 0 | 0 | 0 | 0 |
| Cheilinus oxycephalus | 0 | 0 | 0 | 0 | 182404 | 6788 | 6902 | 0 | 0 | 0 | 0 | 0 | 0 | 0 | 0 | 0 | 0 | 0 |
| Cheilinus trilobatus | 9384 | 64983 | 337404 | 91896 | 783 | 43 | 47 | 2762 | 0 | 2232 | 0 | 0 | 0 | 0 | 111498 | 0 | 0 | 0 |
| Chromis | 347721 | 688014 | 151167 | 1980872 | 1638960 | 194546 | 327712 | 279536 | 2602946 | 1918 | 0 | 9620 | 2635 | 1151704 | 80770 | 56675 | 0 | 0 |
| Chromis viridis | 92 | 0 | 0 | 0 | 3652 | 124 | 6727 | 17911 | 8096 | 0 | 0 | 0 | 0 | 0 | 0 | 0 | 0 | 0 |
| Chrysiptera | 2124 | 0 | 51 | 434 | 0 | 425 | 4286 | 11791 | 1792 | 0 | 0 | 0 | 0 | 302 | 15 | 0 | 0 | 0 |
| Cichliformes | 0 | 18993 | 0 | 0 | 0 | 12329 | 0 | 0 | 3852 | 0 | 0 | 0 | 0 | 0 | 644633 | 0 | 0 | 0 |
| Cirrhitichthys oxycephalus | 44960 | 20616 | 64356 | 1044 | 0 | 7358 | 0 | 0 | 3364 | 0 | 0 | 0 | 0 | 22552 | 1508 | 0 | 0 | 0 |
| Cirrhitidae | 0 | 0 | 0 | 0 | 0 | 26669 | 0 | 0 | 0 | 0 | 0 | 0 | 0 | 0 | 0 | 1440 | 0 | 0 |
| Clupeiformes | 0 | 0 | 0 | 0 | 80731 | 27402 | 0 | 764 | 3048 | 0 | 0 | 0 | 0 | 0 | 0 | 0 | 0 | 0 |
| Clupeocephala | 52022 | 42320 | 258 | 636 | 935218 | 11853 | 1491 | 8917 | 25270 | 0 | 0 | 0 | 63096 | 120616 | 3505 | 48 | 0 | 0 |
| Coris | 41 | 0 | 190 | 0 | 4365 | 283 | 397 | 4065 | 53 | 280 | 0 | 0 | 0 | 0 | 4078 | 0 | 0 | 0 |
| Coryphaena equiselis | 0 | 0 | 0 | 0 | 0 | 0 | 0 | 0 | 0 | 0 | 0 | 0 | 190 | 0 | 0 | 0 | 0 | 0 |
| Crenimugil crenilabis | 82059 | 203110 | 77440 | 0 | 0 | 167 | 0 | 0 | 0 | 0 | 0 | 0 | 0 | 0 | 0 | 0 | 0 | 0 |
| Crossorhombus valderostratus | 4291 | 15157 | 0 | 0 | 0 | 0 | 0 | 0 | 0 | 0 | 0 | 0 | 0 | 0 | 0 | 0 | 0 | 0 |
| Ctenosquamata | 0 | 0 | 0 | 0 | 0 | 2652 | 0 | 0 | 0 | 0 | 0 | 0 | 0 | 0 | 0 | 0 | 0 | 0 |
| Cubiceps squamiceps | 170 | 718 | 0 | 14834 | 0 | 0 | 0 | 0 | 0 | 1480020 | 3234 | 0 | 18132 | 0 | 1273 | 0 | 0 | 0 |
| Cypriniformes | 265 | 90 | 317 | 0 | 379 | 0 | 12929 | 0 | 0 | 0 | 0 | 0 | 0 | 0 | 0 | 0 | 0 | 0 |
| Cyprinodontiformes | 0 | 0 | 0 | 0 | 0 | 565 | 269 | 0 | 16 | 0 | 0 | 0 | 0 | 0 | 0 | 0 | 0 | 0 |
| Dactyloptena orientalis | 0 | 0 | 0 | 0 | 0 | 0 | 0 | 0 | 0 | 0 | 0 | 0 | 9444 | 0 | 0 | 0 | 0 | 0 |
| Dascyllus | 0 | 0 | 0 | 12924 | 35702 | 8610 | 51428 | 3709 | 114076 | 0 | 0 | 0 | 0 | 0 | 0 | 0 | 0 | 0 |
| Dascyllus trimaculatus | 933 | 3009 | 0 | 0 | 43200 | 554 | 0 | 691 | 5855 | 0 | 0 | 0 | 0 | 0 | 0 | 0 | 0 | 0 |
| Dasyatidae | 0 | 0 | 0 | 0 | 0 | 7805 | 0 | 0 | 0 | 0 | 0 | 0 | 0 | 0 | 0 | 0 | 0 | 0 |
| Decapterus macarellus | 0 | 0 | 339 | 5734 | 2205 | 0 | 0 | 0 | 0 | 0 | 0 | 0 | 0 | 12062 | 0 | 269 | 0 | 0 |
| Diaphus | 126 | 7914 | 10084 | 774 | 320 | 6572 | 0 | 17103 | 4275 | 96700 | 215 | 14663 | 7472 | 19288 | 0 | 20 | 0 | 495 |
| Diaphus splendidus | 0 | 0 | 0 | 0 | 0 | 0 | 0 | 0 | 0 | 0 | 72205 | 0 | 255 | 0 | 0 | 346 | 0 | 0 |
| Diodon hystrix | 369 | 0 | 0 | 0 | 0 | 0 | 0 | 0 | 18 | 0 | 0 | 0 | 263 | 0 | 0 | 0 | 0 | 0 |
| Diplophos | 0 | 0 | 2235 | 0 | 0 | 0 | 0 | 0 | 0 | 0 | 0 | 0 | 0 | 278 | 0 | 1382 | 0 | 9072 |
| Echidna | 0 | 314 | 0 | 0 | 0 | 0 | 0 | 0 | 0 | 0 | 0 | 0 | 0 | 0 | 0 | 7284 | 0 | 0 |
| Elagatis bipinnulata | 0 | 0 | 0 | 0 | 0 | 6386 | 0 | 0 | 0 | 0 | 0 | 1479 | 20542 | 0 | 0 | 0 | 0 | 0 |
| Elasmobranchii | 0 | 0 | 0 | 0 | 0 | 0 | 28 | 0 | 23 | 0 | 0 | 0 | 0 | 0 | 0 | 0 | 0 | 0 |
| Encrasicholina punctifer | 0 | 3450 | 15488 | 462994 | 50949 | 0 | 1091 | 0 | 0 | 0 | 0 | 66300 | 146 | 7451 | 0 | 0 | 0 | 0 |
| Epinephelus | 85 | 228 | 76434 | 0 | 0 | 0 | 28807 | 0 | 0 | 0 | 0 | 0 | 0 | 0 | 0 | 0 | 0 | 0 |
| Epinephelus fuscoguttatus | 0 | 0 | 0 | 180 | 0 | 0 | 0 | 0 | 0 | 0 | 0 | 0 | 0 | 0 | 0 | 0 | 0 | 0 |
| Euacanthomorphacea | 573806 | 93500 | 98153 | 46743 | 176830 | 326328 | 366596 | 74614 | 138866 | 0 | 5786 | 0 | 0 | 21760 | 94470 | 437 | 0 | 0 |
| Eupercaria | 15462 | 3875 | 5778 | 0 | 2496 | 3999 | 10637 | 12730 | 8548 | 0 | 0 | 0 | 0 | 0 | 604 | 0 | 0 | 0 |
| Eurypterygia | 0 | 0 | 0 | 0 | 0 | 0 | 19750 | 0 | 974 | 0 | 0 | 0 | 0 | 0 | 0 | 1244 | 0 | 0 |
| Euteleosteomorpha | 0 | 0 | 0 | 0 | 0 | 0 | 0 | 0 | 0 | 0 | 0 | 0 | 0 | 0 | 0 | 0 | 799 | 0 |
| Exocoetidae | 148 | 0 | 2790 | 431923 | 11 | 16669 | 0 | 5138 | 7164 | 0 | 513683 | 7632602 | 13979258 | 1756 | 0 | 83076 | 241643 | 19 |
| Fistularia commersonii | 182 | 0 | 0 | 0 | 9608 | 940 | 13946 | 0 | 0 | 0 | 8694 | 0 | 0 | 0 | 0 | 0 | 0 | 0 |
| Fowleria vaiulae | 0 | 0 | 0 | 0 | 8480 | 15594 | 0 | 0 | 0 | 0 | 0 | 0 | 0 | 0 | 0 | 0 | 0 | 0 |
| Gempylus serpens | 0 | 0 | 0 | 0 | 0 | 0 | 0 | 549 | 0 | 1348 | 0 | 0 | 90852 | 0 | 0 | 25625 | 43344 | 12 |
| Gnatholepis anjerensis | 351580 | 6913 | 265 | 52761 | 0 | 81612 | 7757 | 8361 | 0 | 0 | 0 | 0 | 0 | 0 | 0 | 0 | 0 | 0 |
| Gobiidae | 1116 | 0 | 0 | 0 | 6962 | 0 | 0 | 0 | 0 | 0 | 0 | 0 | 0 | 0 | 0 | 0 | 0 | 0 |
| Gomphosus | 79629 | 22502 | 94104 | 56436 | 760362 | 40762 | 0 | 16919 | 52213 | 5539 | 8664 | 0 | 0 | 89667 | 619440 | 10665 | 0 | 0 |
| Grammistes sexlineatus | 8318 | 0 | 0 | 0 | 0 | 0 | 0 | 0 | 5828 | 0 | 0 | 0 | 0 | 0 | 0 | 0 | 0 | 0 |
| Gunnellichthys monostigma | 0 | 0 | 0 | 0 | 2016 | 38132 | 13548 | 2513 | 1960 | 0 | 0 | 0 | 0 | 0 | 0 | 0 | 0 | 0 |
| Gymnothorax | 831 | 0 | 7378 | 0 | 0 | 2687 | 0 | 0 | 679 | 0 | 0 | 0 | 0 | 0 | 733 | 100132 | 0 | 0 |
| Gymnothorax flavimarginatus | 18392 | 0 | 10270 | 10979 | 17611 | 0 | 51013 | 14340 | 19326 | 0 | 0 | 0 | 0 | 0 | 8570 | 0 | 0 | 0 |
| Halichoeres | 1053 | 0 | 0 | 4176 | 47143 | 517 | 70007 | 5748 | 4658 | 0 | 0 | 0 | 0 | 0 | 896 | 0 | 0 | 0 |
| Halichoeres hortulanus | 5564 | 0 | 24734 | 0 | 7011 | 975 | 4457 | 0 | 0 | 0 | 34535 | 0 | 0 | 0 | 7003 | 0 | 0 | 0 |
| Halichoeres scapularis | 323961 | 12048 | 158070 | 3680 | 22518 | 68798 | 61856 | 173408 | 45471 | 0 | 0 | 0 | 0 | 4112 | 1204485 | 0 | 0 | 0 |
| Hemigymnus | 0 | 0 | 0 | 0 | 0 | 0 | 0 | 0 | 494 | 0 | 0 | 36053 | 3497 | 0 | 0 | 0 | 0 | 0 |
| Hemiramphidae | 0 | 0 | 31 | 0 | 0 | 0 | 0 | 0 | 0 | 0 | 0 | 0 | 0 | 0 | 0 | 0 | 0 | 0 |
| Hemiramphus | 0 | 0 | 38471 | 20503 | 0 | 0 | 0 | 0 | 13213 | 210 | 0 | 13309 | 685325 | 0 | 0 | 0 | 4744 | 0 |
| Heteroconger hassi | 0 | 0 | 0 | 0 | 1784 | 3124 | 0 | 0 | 0 | 0 | 0 | 0 | 2818 | 0 | 0 | 0 | 0 | 0 |
| Himantura | 0 | 0 | 0 | 0 | 28630 | 297 | 0 | 0 | 265740 | 0 | 0 | 0 | 0 | 0 | 0 | 0 | 0 | 0 |
| Holocentridae | 5400 | 0 | 587 | 5950 | 160467 | 1740 | 0 | 0 | 0 | 0 | 0 | 0 | 3130 | 0 | 0 | 1120 | 0 | 0 |
| Holocentriformes | 410 | 0 | 0 | 0 | 0 | 0 | 0 | 0 | 0 | 0 | 0 | 0 | 0 | 0 | 0 | 0 | 0 | 0 |
| Iniistius | 0 | 0 | 0 | 0 | 21876 | 52 | 0 | 1452 | 2942 | 0 | 0 | 0 | 0 | 0 | 0 | 0 | 0 | 0 |
| Katsuwonus pelamis | 2706 | 0 | 3424 | 16780 | 0 | 180 | 3667 | 0 | 2485 | 33870 | 349536 | 0 | 1528252 | 477 | 0 | 6156 | 115020 | 0 |
| Kyphosus | 21301 | 8835 | 12450 | 5142 | 0 | 0 | 0 | 0 | 0 | 0 | 0 | 0 | 0 | 22182 | 2506 | 0 | 0 | 0 |
| Kyphosus cinerascens | 7656 | 8645 | 5410 | 2646 | 116145 | 100636 | 25634 | 0 | 74331 | 0 | 0 | 0 | 0 | 67669 | 8212 | 0 | 0 | 0 |
| Kyphosus vaigiensis | 21593 | 0 | 47916 | 68223 | 58447 | 11807 | 0 | 0 | 0 | 0 | 0 | 0 | 0 | 0 | 0 | 0 | 0 | 0 |
| Labridae | 489588 | 829917 | 1744737 | 1429087 | 30403 | 20557 | 17730 | 28551 | 133320 | 0 | 127892 | 0 | 0 | 0 | 380044 | 124891 | 6799 | 40 |
| Labriformes | 0 | 0 | 0 | 0 | 257664 | 15672 | 70489 | 0 | 17029 | 0 | 0 | 0 | 0 | 0 | 0 | 0 | 0 | 0 |
| Labroides | 0 | 710 | 17225 | 0 | 0 | 3451 | 0 | 0 | 9714 | 0 | 0 | 0 | 0 | 0 | 6807 | 0 | 0 | 0 |
| Lagocephalus lagocephalus | 0 | 0 | 0 | 0 | 0 | 0 | 11 | 0 | 0 | 0 | 0 | 0 | 0 | 0 | 0 | 0 | 0 | 0 |
| Lethrinus | 12324 | 556 | 0 | 0 | 10348 | 4576 | 0 | 886 | 55882 | 0 | 0 | 0 | 0 | 0 | 1573 | 0 | 0 | 0 |
| Lutjanus | 43799 | 0 | 30801 | 29563 | 460363 | 297356 | 26408 | 43919 | 81209 | 0 | 37509 | 0 | 3584 | 6503 | 4239 | 449781 | 266411 | 33 |
| Macropharyngodon bipartitus | 0 | 13254 | 1967 | 0 | 8875 | 74 | 0 | 6767 | 0 | 0 | 0 | 0 | 0 | 0 | 0 | 84 | 0 | 0 |
| Melichthys | 0 | 0 | 0 | 0 | 0 | 0 | 0 | 0 | 0 | 0 | 17803 | 0 | 0 | 0 | 0 | 4050 | 0 | 0 |
| Mobula tarapacana | 0 | 0 | 0 | 0 | 1206 | 0 | 0 | 0 | 0 | 0 | 0 | 0 | 0 | 0 | 0 | 0 | 0 | 0 |
| Monodactylus | 0 | 1682 | 0 | 11630 | 0 | 0 | 0 | 0 | 0 | 0 | 0 | 0 | 0 | 0 | 0 | 0 | 0 | 0 |
| Monotaxis | 0 | 0 | 0 | 0 | 0 | 0 | 0 | 0 | 16 | 0 | 161315 | 0 | 0 | 0 | 0 | 41 | 0 | 0 |
| Moringua | 0 | 0 | 0 | 0 | 0 | 296 | 0 | 0 | 0 | 0 | 0 | 0 | 0 | 0 | 0 | 40 | 0 | 0 |
| Mugilidae | 0 | 0 | 0 | 0 | 0 | 15 | 0 | 0 | 0 | 0 | 0 | 0 | 0 | 0 | 0 | 0 | 0 | 0 |
| Mugiliformes | 0 | 33 | 0 | 0 | 0 | 0 | 0 | 0 | 3701 | 0 | 0 | 0 | 0 | 0 | 0 | 0 | 34987 | 72576 |
| Mullidae | 0 | 10597 | 1521 | 0 | 4478 | 1662 | 0 | 39256 | 0 | 0 | 0 | 0 | 0 | 0 | 0 | 0 | 0 | 0 |
| Mulloidichthys | 7500 | 654 | 3193 | 0 | 4511 | 1672 | 0 | 0 | 0 | 0 | 0 | 0 | 12411 | 0 | 0 | 11398 | 0 | 0 |
| Muraenidae | 1086 | 115 | 0 | 18031 | 15881 | 16682 | 6649 | 0 | 25174 | 0 | 0 | 0 | 0 | 0 | 0 | 77 | 0 | 0 |
| Myctophidae | 4963 | 9273 | 5456 | 22842 | 6515 | 664 | 0 | 11129 | 8270 | 419676 | 49020 | 0 | 34365 | 71979 | 0 | 75279 | 100676 | 48454 |
| Myctophiformes | 18 | 0 | 0 | 0 | 0 | 0 | 8954 | 0 | 0 | 0 | 0 | 0 | 662 | 67839 | 0 | 532 | 2579 | 12 |
| Myctophum | 0 | 0 | 0 | 0 | 2824 | 18028 | 12 | 5085 | 0 | 0 | 0 | 0 | 6956 | 0 | 0 | 4126 | 0 | 0 |
| Myliobatiformes | 0 | 0 | 0 | 0 | 0 | 0 | 0 | 0 | 246 | 0 | 0 | 0 | 0 | 0 | 0 | 0 | 0 | 0 |
| Myripristis | 25863 | 0 | 0 | 6508 | 65347 | 110670 | 0 | 50938 | 28704 | 0 | 0 | 0 | 65 | 0 | 10510 | 0 | 0 | 0 |
| Myripristis berndti | 468 | 0 | 4699 | 14038 | 10300 | 91 | 0 | 0 | 5186 | 0 | 0 | 0 | 15013 | 870 | 12816 | 0 | 0 | 0 |
| Naso | 146787 | 1073856 | 1021984 | 10358 | 137809 | 226540 | 172949 | 443706 | 111978 | 427815 | 0 | 70497 | 0 | 438659 | 81783 | 357871 | 0 | 3634 |
| Nealotus tripes | 848 | 0 | 0 | 0 | 0 | 0 | 0 | 0 | 0 | 0 | 0 | 0 | 0 | 0 | 0 | 0 | 0 | 0 |
| Nemateleotris | 0 | 0 | 0 | 16000 | 0 | 0 | 0 | 0 | 0 | 0 | 0 | 0 | 0 | 0 | 0 | 0 | 0 | 0 |
| Notoscopelus caudispinosus | 0 | 0 | 0 | 1012 | 0 | 0 | 0 | 0 | 0 | 0 | 0 | 0 | 0 | 0 | 0 | 0 | 0 | 0 |
| Novaculichthys | 2970 | 0 | 113 | 0 | 49 | 2400 | 2009 | 58761 | 729 | 0 | 0 | 0 | 0 | 0 | 58 | 14 | 0 | 0 |
| Odonus niger | 0 | 0 | 0 | 0 | 4335 | 6105 | 6657 | 10734 | 49370 | 113 | 0 | 0 | 0 | 0 | 0 | 0 | 0 | 0 |
| Ophiocara | 1258 | 3678 | 48364 | 0 | 0 | 0 | 0 | 0 | 0 | 0 | 0 | 0 | 0 | 0 | 0 | 0 | 0 | 0 |
| Ostichthys | 0 | 0 | 0 | 0 | 0 | 0 | 1993 | 0 | 0 | 0 | 0 | 0 | 0 | 0 | 0 | 0 | 0 | 37 |
| Ostorhinchus | 59 | 0 | 0 | 0 | 0 | 4056 | 0 | 0 | 37643 | 0 | 0 | 0 | 0 | 0 | 0 | 0 | 0 | 0 |
| Ostorhinchus apogonoides | 0 | 3536 | 5635 | 0 | 140466 | 28584 | 0 | 0 | 1034 | 0 | 0 | 0 | 0 | 0 | 1818 | 0 | 0 | 0 |
| Ostorhinchus taeniophorus | 0 | 0 | 955 | 0 | 0 | 1548 | 0 | 2207 | 40987 | 0 | 0 | 0 | 0 | 0 | 4275 | 0 | 0 | 0 |
| Ostraciidae | 1370 | 0 | 0 | 0 | 0 | 0 | 0 | 0 | 0 | 0 | 0 | 0 | 0 | 0 | 1920 | 0 | 0 | 0 |
| Oxycheilinus | 66 | 0 | 0 | 0 | 4661 | 9714 | 0 | 51678 | 10068 | 0 | 0 | 0 | 0 | 0 | 0 | 0 | 0 | 0 |
| Paracirrhites arcatus | 0 | 0 | 647 | 303 | 3228 | 37130 | 0 | 19216 | 144315 | 0 | 0 | 0 | 0 | 0 | 0 | 10956 | 0 | 0 |
| Parapercis hexophtalma | 0 | 0 | 109 | 0 | 0 | 0 | 0 | 0 | 0 | 0 | 0 | 0 | 0 | 0 | 0 | 0 | 0 | 0 |
| Parexocoetus | 0 | 0 | 0 | 0 | 2581 | 305 | 0 | 34782 | 0 | 0 | 0 | 0 | 0 | 0 | 0 | 0 | 0 | 0 |
| Parupeneus | 78730 | 17037 | 28816 | 76762 | 44304 | 53728 | 76769 | 21835 | 209217 | 365977 | 0 | 0 | 205 | 0 | 0 | 11579 | 6055 | 14 |
| Pastinachus atrus | 0 | 0 | 0 | 0 | 0 | 2549 | 0 | 0 | 0 | 0 | 0 | 0 | 0 | 0 | 0 | 0 | 0 | 0 |
| Pateobatis | 1156 | 10092 | 11837 | 0 | 1456 | 91578 | 47533 | 6003 | 878048 | 0 | 0 | 0 | 0 | 0 | 0 | 0 | 0 | 0 |
| Pempheriformes | 0 | 0 | 0 | 0 | 0 | 0 | 0 | 0 | 0 | 0 | 0 | 0 | 0 | 0 | 0 | 3826 | 0 | 0 |
| Pempheris | 0 | 374 | 0 | 0 | 0 | 13675 | 40689 | 0 | 358 | 119278 | 0 | 0 | 0 | 22022 | 9992 | 0 | 0 | 0 |
| Perciformes | 774 | 23 | 123 | 0 | 0 | 8583 | 0 | 0 | 1155 | 0 | 0 | 0 | 0 | 0 | 0 | 1816 | 0 | 0 |
| Percomorphaceae | 346481 | 344384 | 171130 | 118280 | 292759 | 108104 | 297018 | 71031 | 113057 | 0 | 363613 | 0 | 0 | 97094 | 27417 | 99488 | 0 | 0 |
| Plectorhinchus | 161590 | 116324 | 176064 | 2966 | 10079 | 98948 | 28876 | 2163 | 648 | 0 | 0 | 0 | 0 | 0 | 1787 | 0 | 0 | 0 |
| Plectroglyphidodon | 16386 | 0 | 20257 | 938 | 26246 | 17189 | 0 | 40648 | 4287 | 0 | 0 | 0 | 0 | 17515 | 2134 | 1054 | 0 | 0 |
| Plectroglyphidodon leucozonus | 852 | 0 | 0 | 0 | 0 | 0 | 0 | 0 | 0 | 0 | 0 | 0 | 0 | 7160 | 0 | 679 | 0 | 0 |
| Pleuronectiformes | 1695 | 0 | 0 | 0 | 0 | 0 | 0 | 0 | 0 | 0 | 0 | 0 | 0 | 0 | 0 | 0 | 0 | 0 |
| Pomacanthus imperator | 0 | 0 | 0 | 0 | 0 | 0 | 106149 | 0 | 0 | 0 | 0 | 0 | 0 | 0 | 0 | 0 | 0 | 0 |
| Pomacentridae | 22253 | 80293 | 1929 | 18591 | 54436 | 1064 | 17779 | 18437 | 35247 | 6140 | 7355 | 0 | 0 | 314 | 6280 | 410594 | 0 | 13623 |
| Pristiapogon | 0 | 0 | 0 | 0 | 1377 | 566 | 0 | 1342 | 710 | 0 | 0 | 0 | 0 | 0 | 0 | 0 | 0 | 0 |
| Pristiapogon kallopterus | 30 | 98 | 0 | 0 | 0 | 2762 | 174 | 0 | 0 | 0 | 0 | 0 | 0 | 0 | 0 | 0 | 0 | 0 |
| Prognichthys sealei | 0 | 0 | 0 | 0 | 0 | 0 | 0 | 0 | 0 | 0 | 0 | 0 | 54949 | 0 | 0 | 0 | 0 | 0 |
| Pseudanthias | 26800 | 6918 | 71344 | 182025 | 716489 | 12789 | 38742 | 92906 | 62434 | 0 | 17523 | 0 | 0 | 297216 | 580 | 47844 | 0 | 0 |
| Pseudanthias squamipinnis | 69648 | 35463 | 10111 | 24597 | 141382 | 300570 | 267688 | 34298 | 299400 | 284 | 0 | 0 | 0 | 12007 | 61290 | 341383 | 5545 | 43 |
| Pseudobalistes flavimarginatus | 126370 | 97230 | 32270 | 10966 | 112284 | 13277 | 24255 | 0 | 18971 | 0 | 0 | 0 | 0 | 0 | 1891 | 10486 | 0 | 0 |
| Pseudocheilinus | 13390 | 0 | 15088 | 6692 | 79653 | 84714 | 7569 | 60820 | 5075 | 0 | 0 | 0 | 0 | 10535 | 7534 | 7666 | 0 | 0 |
| Pseudocheilinus evanidus | 0 | 0 | 0 | 1055 | 72654 | 36 | 10929 | 0 | 273 | 0 | 0 | 0 | 0 | 0 | 0 | 0 | 0 | 0 |
| Pseudocoris | 0 | 0 | 0 | 0 | 0 | 867 | 58 | 688 | 0 | 0 | 87 | 0 | 0 | 0 | 0 | 0 | 0 | 0 |
| Ptereleotris | 3288 | 0 | 0 | 4976 | 37350 | 35860 | 14878 | 456 | 14767 | 0 | 0 | 0 | 0 | 3143 | 4055 | 70878 | 0 | 0 |
| Ptereleotris heteroptera | 0 | 0 | 0 | 0 | 11823 | 0 | 0 | 0 | 624 | 0 | 0 | 63104 | 0 | 0 | 0 | 0 | 0 | 0 |
| Pterocaesio | 0 | 0 | 46 | 0 | 0 | 0 | 0 | 0 | 0 | 0 | 0 | 0 | 0 | 0 | 0 | 14707 | 0 | 0 |
| Pterocaesio tile | 31738 | 1096 | 19827 | 96160 | 12389 | 3298 | 290 | 7282 | 1792 | 0 | 0 | 0 | 0 | 49734 | 6756 | 156209 | 0 | 0 |
| Pygoplites diacanthus | 0 | 0 | 4253 | 0 | 7042 | 0 | 0 | 0 | 0 | 0 | 0 | 0 | 0 | 0 | 0 | 0 | 0 | 0 |
| Rhabdamia | 0 | 0 | 0 | 0 | 337 | 12216 | 0 | 19964 | 788 | 0 | 0 | 0 | 0 | 0 | 0 | 0 | 0 | 0 |
| Rhinecanthus | 74370 | 117 | 14514 | 461 | 0 | 3819 | 44037 | 0 | 3215 | 0 | 0 | 0 | 0 | 13550 | 0 | 53826 | 0 | 0 |
| Rhinecanthus aculeatus | 114256 | 67836 | 225662 | 36768 | 41700 | 12037 | 82755 | 1299 | 124626 | 0 | 0 | 0 | 0 | 2494 | 751 | 108 | 0 | 0 |
| Salarias | 2804 | 0 | 495 | 0 | 0 | 0 | 0 | 0 | 0 | 0 | 0 | 0 | 0 | 0 | 0 | 0 | 0 | 0 |
| Salmoniformes | 0 | 0 | 0 | 0 | 0 | 48 | 0 | 0 | 0 | 0 | 0 | 0 | 77 | 0 | 0 | 0 | 0 | 0 |
| Sargocentron | 1796 | 0 | 2428 | 3123 | 0 | 1258 | 0 | 0 | 26338 | 0 | 0 | 0 | 0 | 0 | 0 | 0 | 0 | 0 |
| Sargocentron diadema | 0 | 0 | 19 | 0 | 42725 | 20884 | 0 | 0 | 1501 | 0 | 0 | 0 | 52190 | 0 | 0 | 0 | 0 | 0 |
| Sargocentron spiniferum | 5995 | 4554 | 2515 | 0 | 0 | 487 | 0 | 0 | 0 | 0 | 0 | 0 | 0 | 0 | 0 | 660 | 0 | 0 |
| Scarus rubroviolaceus | 480312 | 50799 | 106464 | 98210 | 252240 | 126977 | 38548 | 194388 | 292406 | 0 | 219074 | 0 | 0 | 85308 | 234520 | 4259 | 0 | 0 |
| Scombridae | 0 | 0 | 0 | 0 | 0 | 0 | 0 | 0 | 0 | 0 | 0 | 0 | 3057 | 246 | 0 | 0 | 0 | 0 |
| Scopelosaurus | 0 | 0 | 0 | 0 | 0 | 0 | 0 | 2342 | 0 | 0 | 0 | 0 | 0 | 0 | 0 | 0 | 0 | 12 |
| Scuticaria tigrina | 0 | 0 | 0 | 0 | 0 | 5114 | 0 | 0 | 0 | 0 | 0 | 0 | 0 | 0 | 0 | 0 | 0 | 0 |
| Serranidae | 0 | 0 | 0 | 5234 | 2526 | 1325 | 0 | 0 | 0 | 0 | 0 | 0 | 0 | 0 | 3730 | 839 | 0 | 0 |
| Siganus | 0 | 0 | 0 | 0 | 0 | 978 | 0 | 0 | 0 | 0 | 0 | 0 | 0 | 0 | 0 | 0 | 0 | 0 |
| Siluriformes | 12 | 0 | 68 | 0 | 0 | 0 | 0 | 0 | 0 | 0 | 0 | 0 | 0 | 0 | 0 | 0 | 0 | 0 |
| Sphyraena | 1576 | 0 | 0 | 0 | 0 | 0 | 0 | 0 | 15 | 0 | 0 | 0 | 0 | 0 | 0 | 0 | 0 | 0 |
| Sphyraenidae | 0 | 0 | 0 | 0 | 762 | 12 | 0 | 0 | 0 | 0 | 0 | 0 | 0 | 0 | 0 | 0 | 0 | 0 |
| Sphyrna sp. MV-2009 | 0 | 0 | 0 | 34 | 0 | 0 | 0 | 0 | 0 | 0 | 0 | 0 | 0 | 0 | 0 | 0 | 0 | 0 |
| Spratelloides | 0 | 3560 | 0 | 0 | 115850 | 637227 | 60181 | 258228 | 256028 | 0 | 0 | 0 | 0 | 0 | 0 | 0 | 0 | 0 |
| Stethojulis | 306632 | 331299 | 897227 | 346832 | 30432 | 42066 | 5973 | 27214 | 86969 | 0 | 0 | 0 | 0 | 62953 | 152625 | 0 | 0 | 0 |
| Stomias | 0 | 0 | 0 | 0 | 0 | 0 | 0 | 0 | 0 | 0 | 0 | 0 | 0 | 0 | 0 | 0 | 163 | 19 |
| Stomiidae | 0 | 0 | 0 | 3100 | 0 | 0 | 0 | 0 | 0 | 0 | 0 | 0 | 0 | 0 | 0 | 0 | 0 | 0 |
| Sufflamen chrysopterum | 0 | 403 | 0 | 0 | 43544 | 302 | 3170 | 0 | 906 | 0 | 0 | 0 | 0 | 0 | 0 | 4400 | 0 | 0 |
| Syngnathiformes | 0 | 0 | 0 | 0 | 21151 | 0 | 11670 | 22034 | 3244 | 0 | 0 | 0 | 0 | 0 | 0 | 0 | 0 | 0 |
| Synodontidae | 0 | 0 | 0 | 0 | 169 | 72 | 0 | 0 | 4973 | 0 | 0 | 0 | 0 | 0 | 0 | 0 | 0 | 0 |
| Synodus variegatus | 96 | 0 | 0 | 0 | 37641 | 43 | 710 | 0 | 6610 | 0 | 0 | 0 | 0 | 3601 | 0 | 0 | 0 | 0 |
| Taeniura | 0 | 0 | 0 | 0 | 9933 | 2641 | 0 | 2901 | 2694 | 0 | 0 | 0 | 0 | 0 | 0 | 0 | 0 | 0 |
| Teleostei | 0 | 0 | 0 | 14418 | 0 | 0 | 0 | 0 | 0 | 0 | 0 | 0 | 0 | 0 | 0 | 0 | 0 | 0 |
| Tetraodontiformes | 0 | 0 | 0 | 0 | 0 | 0 | 0 | 996 | 55 | 0 | 0 | 0 | 0 | 0 | 0 | 0 | 0 | 0 |
| Thalassoma | 417705 | 565466 | 1032262 | 385602 | 329014 | 338841 | 28634 | 46175 | 134181 | 260 | 0 | 0 | 0 | 706542 | 961577 | 97393 | 0 | 0 |
| Thalassoma amblycephalum | 697086 | 851829 | 648216 | 819196 | 19750 | 10528 | 87180 | 0 | 8860 | 0 | 0 | 0 | 0 | 6178068 | 1937773 | 115724 | 40198 | 2973522 |
| Thalassoma hardwicke | 242472 | 65428 | 233177 | 23688 | 6177 | 23305 | 0 | 0 | 1184 | 0 | 0 | 0 | 0 | 61008 | 5267883 | 0 | 0 | 0 |
| Thunnus | 0 | 0 | 0 | 0 | 0 | 1112 | 0 | 0 | 0 | 0 | 170502 | 80082 | 8317980 | 0 | 0 | 11880 | 9088 | 12 |
| Triphoturus | 0 | 22601 | 0 | 0 | 0 | 0 | 0 | 0 | 0 | 3424 | 0 | 0 | 24861 | 18214 | 0 | 271 | 0 | 13 |
| Tylosurus | 5604 | 1156 | 23370 | 182869 | 241110 | 26205 | 44 | 0 | 93702 | 0 | 38 | 24 | 287596 | 14809 | 215 | 11930 | 0 | 0 |
| Tylosurus acus | 591 | 92601 | 18980 | 250704 | 498412 | 60641 | 22040 | 83683 | 16402 | 0 | 442406 | 2629528 | 254244 | 23244 | 6564 | 380686 | 1850470 | 353112 |
| Uranoscopidae | 0 | 0 | 0 | 0 | 0 | 0 | 0 | 0 | 0 | 0 | 0 | 0 | 10377 | 0 | 0 | 0 | 0 | 0 |
| Urogymnus asperrimus | 0 | 0 | 0 | 0 | 81506 | 317541 | 60414 | 20052 | 827372 | 0 | 0 | 0 | 0 | 0 | 0 | 0 | 0 | 0 |
| Valenciennea | 0 | 0 | 0 | 0 | 1182 | 1044 | 0 | 0 | 0 | 0 | 0 | 0 | 0 | 0 | 159 | 0 | 0 | 0 |
| Variola | 0 | 0 | 0 | 0 | 5698 | 0 | 0 | 1486 | 0 | 0 | 0 | 0 | 0 | 0 | 0 | 0 | 0 | 0 |
| Wetmorella | 0 | 0 | 0 | 0 | 14 | 0 | 0 | 0 | 1011 | 0 | 0 | 0 | 0 | 0 | 0 | 0 | 0 | 0 |
| Zanclus cornutus | 1504 | 0 | 0 | 0 | 3451 | 31987 | 109754 | 61 | 57013 | 0 | 72432 | 0 | 4992 | 0 | 0 | 737 | 0 | 0 |
| Zebrasoma | 0 | 0 | 0 | 0 | 76587 | 0 | 0 | 0 | 0 | 0 | 0 | 0 | 0 | 0 | 5692 | 0 | 0 | 0 |
| Zebrasoma desjardinii | 0 | 0 | 0 | 0 | 0 | 2868 | 0 | 0 | 0 | 0 | 0 | 0 | 0 | 0 | 0 | 0 | 0 | 0 |

**Table S3:** Number of species present and sequenced along with their sequencing percentage based on the TAAF faunistic list. These results are present by orders found in the scattered islands.

| Order | Number of species | Number of species sequenced | Percentage of species sequenced |
| --- | --- | --- | --- |
| Albuliformes | 1 | 1 | 100 |
| Anguilliformes | 19 | 7 | 37 |
| Atheriniformes | 2 | 1 | 50 |
| Aulopiformes | 7 | 3 | 42.8 |
| Beloniformes | 7 | 3 | 42.8 |
| Beryciformes | 20 | 3 | 15 |
| Carcharhiniformes | 16 | 12 | 75 |
| Clupeiformes | 3 | 2 | 66.7 |
| Gonorhynchiformes | 1 | 1 | 100 |
| Lamniformes | 4 | 4 | 100 |
| Lophiiformes | 1 | 0 | 0 |
| Mugiliformes | 1 | 1 | 100 |
| Myctophiformes | 2 | 0 | 0 |
| Myliobatiformes | 10 | 7 | 70 |
| Ophidiiformes | 4 | 0 | 0 |
| Orectolobiformes | 3 | 3 | 100 |
| Perciformes | 558 | 233 | 41.8 |
| Pleuronectiformes | 1 | 0 | 0 |
| Scorpaeniformes | 16 | 3 | 18.7 |
| Stomiiformes | 2 | 0 | 0 |
| Syngnathiformes | 4 | 2 | 50 |
| Tetraodontiformes | 42 | 23 | 54. 8 |
| Torpediniformes | 1 | 0 | 0 |

**Supplementary Material 3: Spatial variation of β diversity**

**Table S4:** Results of Gaussian generalised linear mixed models (GLMM), with spatial distance and island identity as an explanatory variable, similarity as a dependent variable, and transect identity as a random effect. We fitted one model for overall MOTU richness and one for each habitat type (benthic and pelagic). We assessed the significance of the relationships by interpreting the p-value. Significant terms are in bold.

|  | Estimate | Lower 95% CI | Upper 95% CI | P value |
| --- | --- | --- | --- | --- |
| All |  |  |  |  |
| Intercept | 0.82 | 0.40 | 1.29 | **<0.001** |
| Distance | -58.29 | -102.30 | -14.64 | **0.016** |
| Island: Glorieuse | -0.13 | -0.57 | 0.33 | 0.526 |
| Island: Juan de Nova | -0.23 | -0.67 | 0.26 | 0.294 |
| Island: Tromelin | -0.15 | -0.71 | 0.31 | 0.512 |
| Benthic/demersal |  |  |  |  |
| Intercept | 0.70 | 0.25 | 1.14 | **0.006** |
| Distance | -57.74 | -95.59 | -12.10 | **0.022** |
| Island: Glorieuse | -0.065 | -0.51 | 0.44 | 0.8 |
| Island: Juan de Nova | -0.093 | -0.64 | 0.36 | 0.7 |
| Island: Tromelin | 0.0012 | -0.61 | 0.52 | 0.98 |
| Pelagic |  |  |  |  |
| Intercept | 0.69 | 0.29 | 1.17 | **0.008** |
| Distance | -56.91 | -98.66 | -13.76 | **0.014** |
| Island: Glorieuse | -0.048 | -0.49 | 0.42 | 0.8 |
| Island: Juan de Nova | -0.084 | -0.56 | 0.36 | 0.71 |
| Island: Tromelin | -0.0092 | -0.52 | 0.48 | 0.96 |

# **Supplementary Material 4: Joint species distribution modelling**


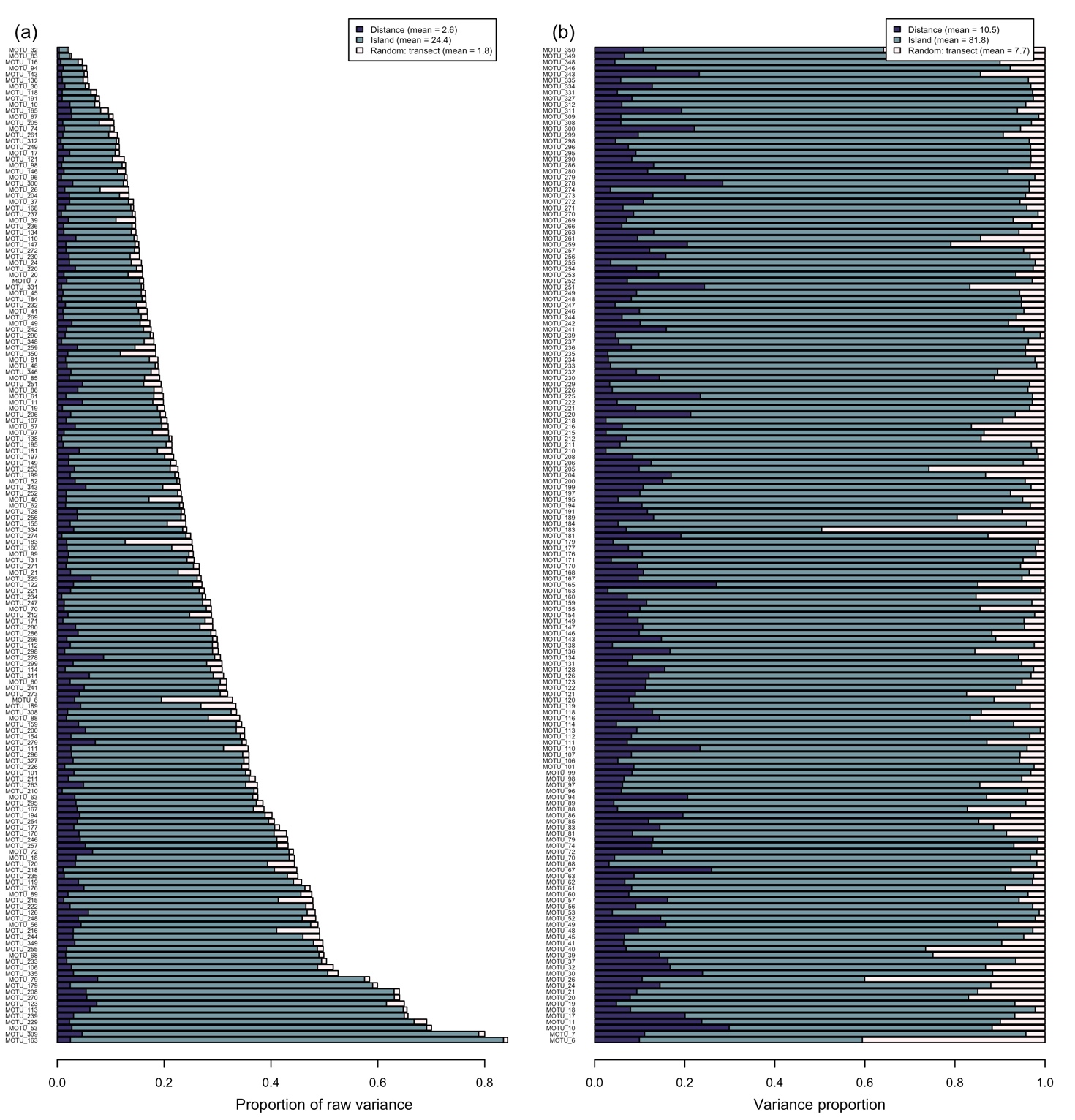


**Figure S4:** Variance partitioning among the explanatory variables included in the models (hierarchical modelling of species communities). Proportion of (a) raw and (b) explained variance explained by each predictor variable. In (a), the order of the bars corresponds to the explanatory power achieved (lowest at the top).

**Table S5:** Beta parameters estimated by the models (hierarchical modelling of species communities. For each of the 143 MOTUs included in the models, we show the estimated median and the 0.975 and 0.025 quantiles of the estimated beta parameters. We multiplied all beta parameters by 100 to interpret them as distance coefficients estimating changes in the probability of occurrence per 100 metres.

| **Family** | **Genus** | **Species** | **Median** | **0.975 quantile** | **0.025 quantile** |
| --- | --- | --- | --- | --- | --- |
| Acanthuridae | Acanthurus | *Acanthurus lineatus* | -0.071 | 0.036 | -0.184 |
| Acanthuridae | Acanthurus | *Acanthurus nigrofuscus* | -0.047 | 0.058 | -0.155 |
| Acanthuridae | Acanthurus |  | -0.063 | 0.060 | -0.192 |
| Acanthuridae | Acanthurus |  | -0.091 | 0.018 | -0.212 |
| Acanthuridae | Acanthurus |  | -0.065 | 0.052 | -0.186 |
| Acanthuridae | Acanthurus |  | -0.122 | 0.004 | -0.259 |
| Acanthuridae | Acanthurus |  | -0.090 | 0.034 | -0.224 |
| Acanthuridae | Naso |  | -0.143 | -0.021 | -0.281 |
| Acanthuridae | Naso |  | -0.132 | -0.012 | -0.267 |
| Acanthuridae | Naso |  | -0.117 | 0.002 | -0.247 |
| Acanthuridae |  |  | -0.075 | 0.040 | -0.193 |
| Acanthuridae |  |  | 0.022 | 0.146 | -0.105 |
| Acanthuridae |  |  | -0.088 | 0.027 | -0.207 |
| Zanclidae | Zanclus | *Zanclus cornutus* | -0.023 | 0.083 | -0.131 |
| Albulidae | Albula |  | -0.091 | 0.034 | -0.221 |
| Muraenidae | Gymnothorax | *Gymnothorax flavimarginatus* | -0.027 | 0.087 | -0.146 |
| Synodontidae | Synodus | *Synodus variegatus* | -0.107 | 0.017 | -0.242 |
| Belonidae | Tylosurus | *Tylosurus acus* | 0.037 | 0.161 | -0.076 |
| Belonidae | Tylosurus |  | -0.062 | 0.050 | -0.184 |
| Belonidae |  |  | -0.060 | 0.052 | -0.176 |
| Exocoetidae |  |  | 0.068 | 0.179 | -0.031 |
| Exocoetidae |  |  | 0.088 | 0.204 | -0.022 |
| Hemiramphidae | Hemiramphus |  | 0.065 | 0.179 | -0.035 |
| Carangidae | Caranx | *Caranx melampygus* | -0.124 | -0.008 | -0.260 |
| Carangidae | Decapterus | *Decapterus macarellus* | -0.004 | 0.108 | -0.117 |
| Carangidae | Elagatis | *Elagatis bipinnulata* | 0.046 | 0.159 | -0.067 |
| Carcharhinidae | Carcharhinus |  | -0.107 | 0.023 | -0.242 |
| Carcharhinidae |  |  | -0.005 | 0.112 | -0.128 |
| Cirrhitidae | Cirrhitichthys | *Cirrhitichthys oxycephalus* | -0.038 | 0.080 | -0.155 |
| Cirrhitidae | Paracirrhites | *Paracirrhites arcatus* | -0.036 | 0.082 | -0.157 |
| Kyphosidae | Kyphosus | *Kyphosus cinerascens* | -0.070 | 0.050 | -0.192 |
| Kyphosidae | Kyphosus | *Kyphosus vaigiensis* | -0.049 | 0.064 | -0.174 |
| Kyphosidae | Kyphosus |  | -0.010 | 0.114 | -0.133 |
| Chaetodontidae | Chaetodon |  | -0.042 | 0.067 | -0.153 |
| Clupeidae | Spratelloides |  | -0.067 | 0.058 | -0.201 |
| Engraulidae | Encrasicholina | *Encrasicholina punctifer* | 0.066 | 0.173 | -0.038 |
| Gobiidae | Gnatholepis | *Gnatholepis anjerensis* | 0.023 | 0.137 | -0.090 |
| Gobiidae | Gunnellichthys | *Gunnellichthys monostigma* | -0.064 | 0.067 | -0.200 |
| Gobiidae | Ptereleotris |  | -0.089 | 0.022 | -0.204 |
| Gobiidae | Ptereleotris |  | -0.055 | 0.063 | -0.174 |
| Chanidae | Chanos | *Chanos chanos* | -0.051 | 0.064 | -0.173 |
| Holocentridae | Myripristis | *Myripristis berndti* | 0.027 | 0.129 | -0.078 |
| Holocentridae | Myripristis |  | -0.024 | 0.079 | -0.124 |
| Holocentridae | Sargocentron | *Sargocentron diadema* | 0.027 | 0.136 | -0.086 |
| Holocentridae | Sargocentron | *Sargocentron spiniferum* | -0.055 | 0.063 | -0.180 |
| Holocentridae | Sargocentron |  | -0.034 | 0.084 | -0.154 |
| Holocentridae |  |  | -0.024 | 0.082 | -0.131 |
| Apogonidae | Ostorhinchus | *Ostorhinchus apogonoides* | -0.079 | 0.040 | -0.206 |
| Apogonidae | Ostorhinchus | *Ostorhinchus taeniophorus* | -0.058 | 0.063 | -0.182 |
| Apogonidae | Pristiapogon | *Pristiapogon kallopterus* | -0.064 | 0.056 | -0.192 |
| Apogonidae | Pristiapogon |  | -0.043 | 0.088 | -0.173 |
| Apogonidae | Rhabdamia |  | -0.079 | 0.046 | -0.219 |
| Labridae | Cheilinus | *Cheilinus trilobatus* | -0.014 | 0.092 | -0.129 |
| Labridae | Coris |  | -0.071 | 0.049 | -0.201 |
| Labridae | Coris |  | -0.035 | 0.085 | -0.162 |
| Labridae | Gomphosus |  | -0.092 | 0.021 | -0.212 |
| Labridae | Halichoeres | *Halichoeres hortulanus* | -0.104 | 0.010 | -0.235 |
| Labridae | Halichoeres | *Halichoeres scapularis* | -0.100 | 0.022 | -0.226 |
| Labridae | Halichoeres |  | -0.142 | -0.011 | -0.288 |
| Labridae | Iniistius |  | -0.079 | 0.049 | -0.218 |
| Labridae | Labroides |  | -0.049 | 0.068 | -0.172 |
| Labridae | Macropharyngodon | *Macropharyngodon bipartitus* | -0.035 | 0.078 | -0.155 |
| Labridae | Novaculichthys |  | -0.117 | 0.001 | -0.243 |
| Labridae | Oxycheilinus |  | -0.078 | 0.050 | -0.209 |
| Labridae | Pseudocheilinus | *Pseudocheilinus evanidus* | -0.051 | 0.065 | -0.175 |
| Labridae | Pseudocheilinus |  | -0.109 | 0.007 | -0.234 |
| Labridae | Pseudocoris |  | -0.047 | 0.073 | -0.182 |
| Labridae | Scarus | *Scarus rubroviolaceus* | -0.066 | 0.050 | -0.188 |
| Labridae | Stethojulis |  | -0.068 | 0.066 | -0.204 |
| Labridae | Thalassoma | *Thalassoma amblycephalum* | -0.077 | 0.042 | -0.204 |
| Labridae | Thalassoma | *Thalassoma hardwicke* | -0.113 | 0.005 | -0.239 |
| Labridae | Thalassoma |  | -0.065 | 0.051 | -0.187 |
| Labridae |  |  | -0.017 | 0.103 | -0.142 |
| Labridae |  |  | -0.008 | 0.112 | -0.125 |
| Labridae |  |  | -0.076 | 0.030 | -0.186 |
| Haemulidae | Plectorhinchus |  | -0.022 | 0.091 | -0.137 |
| Lutjanidae | Caesio | *Caesio teres* | -0.041 | 0.067 | -0.152 |
| Lutjanidae | Caesio |  | -0.139 | -0.023 | -0.275 |
| Lutjanidae | Caesio |  | -0.033 | 0.077 | -0.149 |
| Lutjanidae | Caesio |  | -0.110 | 0.023 | -0.253 |
| Lutjanidae | Caesio |  | -0.047 | 0.072 | -0.169 |
| Lutjanidae | Lutjanus |  | -0.006 | 0.097 | -0.109 |
| Lutjanidae | Lutjanus |  | -0.102 | 0.020 | -0.240 |
| Lutjanidae | Lutjanus |  | -0.112 | 0.009 | -0.246 |
| Lutjanidae | Lutjanus |  | -0.012 | 0.093 | -0.115 |
| Lutjanidae | Pterocaesio | *Pterocaesio tile* | -0.083 | 0.029 | -0.201 |
| Mugilidae | Crenimugil | *Crenimugil crenilabis* | -0.052 | 0.067 | -0.174 |
| Myctophidae | Benthosema |  | -0.089 | 0.039 | -0.221 |
| Myctophidae | Bolinichthys |  | -0.005 | 0.103 | -0.109 |
| Myctophidae | Bolinichthys |  | -0.075 | 0.042 | -0.199 |
| Myctophidae | Diaphus |  | 0.047 | 0.149 | -0.051 |
| Myctophidae | Myctophum |  | -0.019 | 0.096 | -0.128 |
| Myctophidae | Triphoturus |  | 0.008 | 0.116 | -0.101 |
| Myctophidae |  |  | -0.005 | 0.099 | -0.117 |
| Myctophidae |  |  | 0.009 | 0.115 | -0.105 |
| Dasyatidae | Pateobatis |  | -0.091 | 0.030 | -0.220 |
| Dasyatidae | Urogymnus | *Urogymnus asperrimus* | -0.100 | 0.037 | -0.245 |
| Potamotrygonidae | Taeniura |  | -0.069 | 0.061 | -0.204 |
| Pempheridae | Pempheris |  | -0.076 | 0.040 | -0.214 |
| Serranidae | Aethaloperca | *Aethaloperca rogaa* | -0.040 | 0.064 | -0.148 |
| Serranidae | Cephalopholis | *Cephalopholis argus* | -0.023 | 0.094 | -0.144 |
| Serranidae | Cephalopholis | *Cephalopholis urodeta* | -0.066 | 0.051 | -0.186 |
| Serranidae | Cephalopholis |  | -0.014 | 0.102 | -0.132 |
| Serranidae | Pseudanthias | *Pseudanthias squamipinnis* | -0.088 | 0.021 | -0.205 |
| Serranidae | Pseudanthias |  | -0.096 | 0.015 | -0.218 |
| Gempylidae | Gempylus | *Gempylus serpens* | 0.012 | 0.118 | -0.101 |
| Nomeidae | Cubiceps | *Cubiceps squamiceps* | 0.030 | 0.139 | -0.079 |
| Scombridae | Katsuwonus | *Katsuwonus pelamis* | 0.022 | 0.125 | -0.082 |
| Scombridae | Thunnus |  | 0.035 | 0.150 | -0.076 |
| Lethrinidae | Lethrinus |  | -0.107 | 0.015 | -0.240 |
| Fistulariidae | Fistularia | *Fistularia commersonii* | -0.091 | 0.027 | -0.221 |
| Mullidae | Mulloidichthys |  | -0.020 | 0.091 | -0.130 |
| Mullidae | Parupeneus |  | -0.074 | 0.037 | -0.196 |
| Mullidae | Parupeneus |  | 0.006 | 0.110 | -0.093 |
| Mullidae | Parupeneus |  | -0.055 | 0.051 | -0.170 |
| Mullidae |  |  | -0.003 | 0.113 | -0.122 |
| Balistidae | Balistapus | *Balistapus undulatus* | -0.067 | 0.044 | -0.182 |
| Balistidae | Odonus | *Odonus niger* | -0.050 | 0.073 | -0.183 |
| Balistidae | Pseudobalistes | *Pseudobalistes flavimarginatus* | -0.096 | 0.019 | -0.218 |
| Balistidae | Rhinecanthus | *Rhinecanthus aculeatus* | -0.099 | 0.014 | -0.221 |
| Balistidae | Rhinecanthus |  | -0.085 | 0.026 | -0.201 |
| Balistidae | Sufflamen | *Sufflamen chrysopterum* | -0.085 | 0.033 | -0.212 |
| Monacanthidae | Cantherhines | *Cantherhines pardalis* | 0.015 | 0.118 | -0.092 |
| Tetraodontidae | Arothron |  | -0.068 | 0.055 | -0.196 |
| Tetraodontidae | Canthigaster |  | -0.008 | 0.106 | -0.123 |
| Pomacanthidae | Centropyge |  | -0.083 | 0.027 | -0.200 |
| Pomacentridae | Abudefduf | *Abudefduf septemfasciatus* | -0.047 | 0.072 | -0.171 |
| Pomacentridae | Abudefduf |  | -0.121 | -0.007 | -0.246 |
| Pomacentridae | Chromis | *Chromis viridis* | -0.082 | 0.042 | -0.216 |
| Pomacentridae | Chromis |  | -0.022 | 0.086 | -0.134 |
| Pomacentridae | Chromis |  | -0.058 | 0.055 | -0.177 |
| Pomacentridae | Chromis |  | -0.020 | 0.095 | -0.136 |
| Pomacentridae | Chromis |  | 0.003 | 0.113 | -0.106 |
| Pomacentridae | Chromis |  | 0.015 | 0.131 | -0.105 |
| Pomacentridae | Chromis |  | -0.049 | 0.058 | -0.160 |
| Pomacentridae | Chrysiptera |  | -0.080 | 0.036 | -0.207 |
| Pomacentridae | Dascyllus | *Dascyllus trimaculatus* | -0.066 | 0.055 | -0.195 |
| Pomacentridae | Dascyllus |  | -0.038 | 0.085 | -0.160 |
| Pomacentridae | Plectroglyphidodon |  | -0.060 | 0.053 | -0.181 |
| Pomacentridae | Plectroglyphidodon |  | -0.096 | 0.022 | -0.233 |
| Pomacentridae |  |  | -0.079 | 0.024 | -0.197 |
| Pomacentridae |  |  | -0.056 | 0.060 | -0.185 |
| Pomacentridae |  |  | -0.042 | 0.078 | -0.167 |
